# Are you afraid? Neurophysiological responses to multidimensional fear in an interactive semi-immersive environment

**DOI:** 10.3389/fnsys.2026.1908489

**Published:** 2026-09-17

**Authors:** Milton O. Candela, J. Emiliano Calderón-Gurubel, Alexandro A. Ortiz-Espinoza, Josemaria Elizondo-Garcia, Carol S. Vélez-Saboyá, Jorge De-J. Lozoya-Santos, Ignacio Rodriguez-Sanchez, Manuel Cebral-Loureda, Mauricio A. Ramírez-Moreno

**Affiliations:** 1 Tecnologico de Monterrey, School of Engineering and Sciences, Monterrey, Mexico; 2 Tecnologico de Monterrey, School of Humanities and Education, Monterrey, Mexico; 3 Universidad Autonoma de Queretaro, Facultad de Lenguas y Letras, Santiago de Querétaro, Mexico

**Keywords:** autonomic, BVP/HRV, EDA, EEG, emotion, fear, neural, psychophysiology

## Abstract

**Introduction:**

Objective fear recognition is crucial for clinical evaluation and understanding emotional processing. This study uses a multi-modal biophysical approach (EEG, HRV, EDA) within an interactive, semi-immersive environment to investigate coordinated brain-body responses across multidimensional fear scenarios.

**Methods:**

Ten participants experienced four fear-related scenes mapped to Kantian cognitive dimensions (Embodied, Conceptual, Discursive, Integrative) featuring real-time adaptive audiovisuals. EEG, HRV, and EDA metrics were concurrently recorded and normalized to a resting baseline to evaluate shared signatures, scene-specific modulations, and cross-modal correlations.

**Results:**

Neural and autonomic fear markers included frontal and parietal activation, elevated EEG-derived indices (Fatigue, Excitement, Engagement), and increased heart rate and skin conductance. Contextual modulation of fear was characterized by bottom-up and top-down processing during Embodied and Conceptual fear, respectively. Multi-modal clustering showed modality-specific organization overall, with a weak but significant global EEG-autonomic association (Mantel test), and one validated cross-modal link between HRV (LF/HF) and EDA (SCR_*P*_).

**Conclusion:**

Fear manifested through both shared neurophysiological signatures and context-dependent cognitive-embodied variations. Combining immersive technology with multi-modal biophysical sensing presents an exploratory, ecologically-valid framework for emotion research that can inform future closed-loop, adaptive paradigms.

## 1 Introduction

Fear recognition is critical for both clinical evaluations, such as trauma assessment, and for understanding normative emotional processing (Ayuso-Moreno et al., 2020; Ribas et al., 2018; Russo et al., 2024; Battaglia et al., 2022). As a fundamental defensive state, fear mobilizes the central and peripheral nervous systems to facilitate rapid detection and response to threats, a reaction commonly known as the fight-or-flight mechanism (Cannon, 1915). This state manifests through increased autonomic arousal and altered cortical activity, whose physiological measurement enables objective assessment and distinction between adaptive vigilance and pathological conditions such as anxiety isorders.

Electroencephalography (EEG), in particular, has been widely used to study rapidly changing cognitive processes with high temporal resolution (Berger, 1929; Luck, 2014), including mental fatigue, vocational interest, cognitive load, attention, and emotions (Ramírez-Moreno et al., 2021; Candela-Leal et al., 2025a,b, 2026; Blanco-Ríos et al., 2024). This is primarly because the spectral EEG features are associated with underlying cognitive states, such as: Delta (δ, 1–4 Hz) to deep sleep, theta (θ, 4–8 Hz) to working memory, alpha (α, 8–12 Hz) to attention, beta (β, 13–30 Hz) to executive action, and gamma (γ, 30–100 Hz) to higher-order cognition (Kropotov, 2009). In the current study, only the theta, alpha, and beta bands will be analyzed, given their roles in mental effort (Candela-Leal et al., 2025b; Villafaina et al., 2021b), attention (Benedek et al., 2014; Ray and Cole, 1985; Klimesch et al., 1999; Jensen, 2002; Sauseng et al., 2005; Rihs et al., 2007), and executive action (Lamarche and Ogilvie, 1997; Pineda-Hernández, 2022), respectively.

Complementary to brain measurements, autonomic activity can be assessed through signals regulated by the Autonomic Nervous System (ANS). In particular, heart rate and sweat gland activity enable the extraction of physiological features such as Heart Rate Variability (HRV) and Electrodermal Activity (EDA), which reflect sympathetic and parasympathetic modulation (Wehrwein et al., 2016; Ghiasi et al., 2020). HRV is based on the successive heartbeats variation over a time interval (Thayer et al., 2012), in which the cardiovascular center adjusts the heart rate by shifting between sympathetic and parasympathetic nervous systems (Loewi, 1921; Levy, 1971). A lower level of HRV, sympathetic activation, is related to reduced regulatory capacity to adapt to challenges or cognitive stressors (Shaffer et al., 2014), while higher HRV is an indicator of resilience, behavior flexibility or cognitive load (Beachaine, 2001; Berntson et al., 2008). EDA measures changes in the electrical conductance of the skin resulting from the modulation of sweat gland activity by the sudomotor nerves (Ghiasi et al., 2020); these changes are determined exclusively by the sympathetic nervous system. Therefore, EDA can be used to reliably measure sympathetic function, as well as cognitive arousal (Ghiasi et al., 2020; Posada-Quintero and Chon, 2020). The most salient characteristic of EDA signals is the appearance of Skin Conductance Responses (SCRs), which are phasic components from a sympathetic reaction to stimuli. Besides the analysis of rapid and smooth changes trough SCRs, the Skin Conductance Level (SCL) measures slow shifts in the signal, defined as the overall conductance obtained from the tonic component of EDA. In conjunction, SCRs and SCL assess the subject's response in event-related experiments or tonic stimuli tests, such as changes in condition, workload, or cognitive stress (Posada-Quintero and Chon, 2020; Posada-Quintero and Bolkhovsky, 2019).

At the brain level, human functional Magnetic Resonance Imaging (fMRI) studies have provided insights into neural circuitry related to fear, including the amygdala, hippocampus, and prefrontal cortex (Åhs et al., 2015; Picó-Pérez et al., 2019; Milad and Quirk, 2012). On the other hand, EEG studies have identified frontal (Rezaei et al., 2025; Kim et al., 2019; DeLaRosa et al., 2014; Apicella et al., 2023; Demaree et al., 2005), parietal (Rezaei et al., 2025; Ugalde-Canitrot et al., 2025; Alemayehu et al., 1995; Yaşgüçlükal et al., 2021; Okazaki et al., 2008), temporal (Ribas et al., 2018; Byeon et al., 2023), and occipital (DeLaRosa et al., 2014; Ugalde-Canitrot et al., 2025; Okazaki et al., 2008) lobes as cortical structures related to fear; relating theta (Rezaei et al., 2025; Kim et al., 2019; DeLaRosa et al., 2014), alpha (Russo et al., 2024; Kim et al., 2019), and beta (Ribas et al., 2018; Russo et al., 2024; Rezaei et al., 2025; Kim et al., 2019; Apicella et al., 2023; Okazaki et al., 2008; Byeon et al., 2023; Bălan et al., 2020a) frequency bands. These have different physiological meanings depending on the oscillatory frequency and brain area. For example, in response to threatening images, there is a theta burst in the occipital region, which processes visual information, followed by a theta burst at the frontal region for decision-making and planning (DeLaRosa et al., 2014). Conversely, beta activity in the same region increases in situations that require significant attention and in which a perceptual shift occurs (Rezaei et al., 2025; Kim et al., 2019; Okazaki et al., 2008).

Beyond brain dynamics, autonomic responses have also been used as objective markers of fear, mainly in the study of fear conditioning. In this context, sympathetic arousal is commonly inferred from SCRs (Staib et al., 2015; Castegnetti et al., 2016), i.e., threatening or fear-relevant cues elicit larger and more frequent SCRs than neutral or safe cues in Pavlovian fear conditioning and extinction paradigms (Christopoulos et al., 2019; Marin et al., 2017). However, SCR analysis is limited by its inability to capture parasympatheti, vagal, activity. This limitation is relevant, as fear-conditioned stimuli have been shown to elicit transient heart rate deceleration, a response generally believed to reflect predominantly vagal activation (Castegnetti et al., 2016). Consequently, increasing efforts have focused on the use of HRV as an index of fear conditioning, given its potential to assess sympathetic and parasympathetic activation in response to stimuli (Battaglia et al., 2022; Battaglia and Thayer, 2022).

While traditional neurophysiological studies of fear often rely on highly controlled, artificial laboratory settings (Ghiasi et al., 2020; Thayer et al., 2012; Staib et al., 2015; Castegnetti et al., 2016), immersive and semi-immersive technologies offer more ecologically valid contexts for studying emotion, with semi-immersive environments enabling free expression and movement while also enhancing immersion (Kisker et al., 2021). The effectiveness of Virtual Reality (VR) in eliciting fear is highly modulated by *presence* (Riva et al., 2015), which is the psychological sense of “being there” in the Virtual Environment (VE) (Witmer and Singer, 1998), characterized by spatial presence, involvement, and realness (Schubert, 2003). Presence is enhanced by *immersion* (Diemer et al., 2015), which depends on the equipment's characteristics used to create and display the VR (Gromer et al., 2018), such as display quality and real-time feedback (Slater, 1999), and has been associated with increased frontal beta activity (Tychkov et al., 2023). Therefore, multi-modal interfaces are essential to provide multisensory stimulation, improving fidelity and presence (Cooper et al., 2018; Honegger et al., 2021; Riva et al., 2007); without them, unimodal interfaces often feel hollow and unrealistic (Maggioni et al., 2019; Mishra et al., 2020).

Consequently, given that VR can evoke emotions (Higuera-Trujillo et al., 2017; Chirico and Gaggioli, 2019; Kisker et al., 2021), they are increasingly used in closed-loop phobia therapies. These systems monitor physiological signals to dynamically adapt exposure scenarios in real-time (Bălan et al., 2020a,b). While exposure may acutely induces stress (EDA and HR) during the experience (Moldoveanu et al., 2023), it reduces post-treatment anxiety across various phobias (Bălan et al., 2020b; Diemer et al., 2016), including acrophobia (fear of heights) (Russo et al., 2024; Apicella et al., 2023; Bălan et al., 2020a; Freeman et al., 2018), ophiophobia (fear of snakes) (Toma et al., 2020; Go et al., 2020; Ding et al., 2020), claustrophobia (fear of enclosed spaces) (Rezaei et al., 2025), pyrophobia (fear of fire) (Nazarova et al., 2023; Obukhov et al., 2023) and fear of public speaking therapy (Reeves et al., 2021; Seuling et al., 2024).

The current paper addresses the static settings and unimodal limitations by integrating non-invasive multi-modal neurophysiological (EEG, BVP, EDA) recordings of participants at a real-time interactive (psychophysiological-based) immersive environment, considering an 8-electrode EEG (OpenBCI) at theta, alpha, and beta spectral bands; in addition to a biometric bracelet (Empatica E4) to dynamically modulate the surrounding projection-based VR setting, including visualizations, lighting, and sound. To identify key neurophysiological responses related to fear-based environments, a pairwise analysis with baseline and an interscene analysis with other expressive scenes within the immersive environment is considered. Specifically, addressing the research questions:

1. How do neural and autonomic responses differ between baseline and fear-related scenes in a semi-immersive environment?
2. How do neural and autonomic responses change across different semi-immersive interactive fear-related scenes?

The hypothesis is that participants in the immersive fear scenario will exhibit increased stress-related autonomic responses (EDA, HRV). On the other hand, in all immersive scenes, there should be increased brain activity associated with visual information processing (occipital), planning (central), emotional processing (parietal), and decision-making (frontal). Thus, showing that fear-related semi-immersive scenarios elicit information processing and activate the sympathetic nervous system at both psychological and physiological levels.

The paper is organized as follows: Section 2 describes the participants (Section 2.1), the signal acquisition devices and process (Section 2.2), the experimental protocol followed (Section 2.3), in addition to also detailing the EEG, BVP, and EDA signal pre-processing (Section 2.4) and processing (Section 2.5), as well as the statistical analysis (Section 2.6). Section 3 uses the normalized EEG, HRV, and EDA features to assess shared brain and autonomic activation patterns across scenes (Section 3.1), and to show cross-device relationships (Section 3.2). The most important findings are discussed in Section 4, where Section 4.1 addresses research question (1) and Section 4.2 addresses research question (2). In addition, Section 4.3 adds complementary inter-device findings on both research questions. Finally, Section 4.4 discusses the study's limitations and Section 5 concludes with the relevance of this article.

## 2 Materials and methods

The following sections describe the participants, experimental procedures, and analytical methods used to evaluate the neurophysiological responses associated with the fear experience.

### 2.1 Participants

Participants consisted in 10 healthy adults (M = 4, F = 6), with ages ranging from 18 to 36 years (μ = 25.11, σ = 6.21). Participants signed an informed consent form in accordance with the 1964 Helsinki Declaration. They were informed about the study, their rights, risks, and benefits, and remained in close contact with the research team. The exclusion criteria ruled out participants who were undergoing pharmacological treatment or had cardiovascular or physical conditions that limited their ability to perform the required movements. The experimental research protocol was granted an exemption (protocol number EHE-2023-03) by the Institutional Research Ethics Committee (CIEI) of Tecnologico de Monterrey. The experiment and signal acquisition were made in collaboration with Centro de Estudios Críticos en Cultura Contemporánea (CECRIT), Universidad Autónoma de Queretaro (UAQ). The space measured 4 by 4 m, and data were recorded over 1 week (29-Apr-2024 - 03-May-2024).

### 2.2 Signal acquisition

EEG signals were acquired through the Ultracortex Mark IV (OpenBCI, New York, NY, USA), a dry-electrode reconfigurable headset that includes eight channels: Two at the prefrontal region (Fp1, Fp2), two at the central region (C3, C4), two at the parietal region (P7, P8), and two at the occipital region (O1, O2); in addition to two ear clips on both earlobes. EEG data was measured in microvolts (μV) at a sampling frequency of 250 Hz. OpenBCI GUI (OpenBCI, New York, NY, USA) was used to measure the impedance of electrodes and electrodes were screwed more tight to the participant's scalp until the impedance values on most channels were less than 750 *kΩ* (Romo-De León et al., 2024), indicated by a green electrode color at the interface, this software was also used to transmit EEG scalp data from the Cyton board to the computer through Bluetooth by leveraging the OpenBCI dongle.

Autonomic responses were acquired through the Empatica E4 wristband (Empatica, Cambridge, MA, USA), a wearable device designed for ambulatory physiological monitoring. The E4 is capable of recording EDA at a sampling frequency of 4 Hz, skin temperature at 4 Hz, triaxial acceleration at 32 Hz and Blood Volume Pulse (BVP) at 64 Hz; thereby allowing the derivation of Interbeat Intervals (IBIs) and Heart Rate (HR). Data were transmitted wirelessly in real-time to the host computer via a second dedicated Bluetooth dongle, enabling continuous data acquisition during the experimental protocol.

### 2.3 Experimental protocol

The experimental design (Figure 1) consisted of a learning experience divided into four blocks, referred to as “scenes,” each consisting of specific 3-min tasks. Participants undertook each scene sequentially, starting with a 2-min eyes open baseline resting period, then scene 1, 2 and 3, and ending with scene 4. Each scene was separated by a 1-min washout period, during which the investigator explained the upcoming task, saved the previous recording, and initiated the next. Scenes order was fixed given the constructive nature of the experience, where decisions in earlier scenes (e.g., words chosen in Scene 2) affect subsequent scenes (e.g., words spoken in Scene 3). The scenes' design was based on (Romo-De León et al., 2024, 2026), which is grounded in Kant's philosophy of the Theory of Knowledge and follows a structure of cognitive learning and integrative levels. Each scene had an underlying logic that provided it with a fundamental structure, representing different levels of cognition into which modern philosophy has divided experience, according to Kant, the primary reference in the modern theory of knowledge (Dennis, 2020; Kuplen, 2023; Webb, 2020).

**Figure 1:**
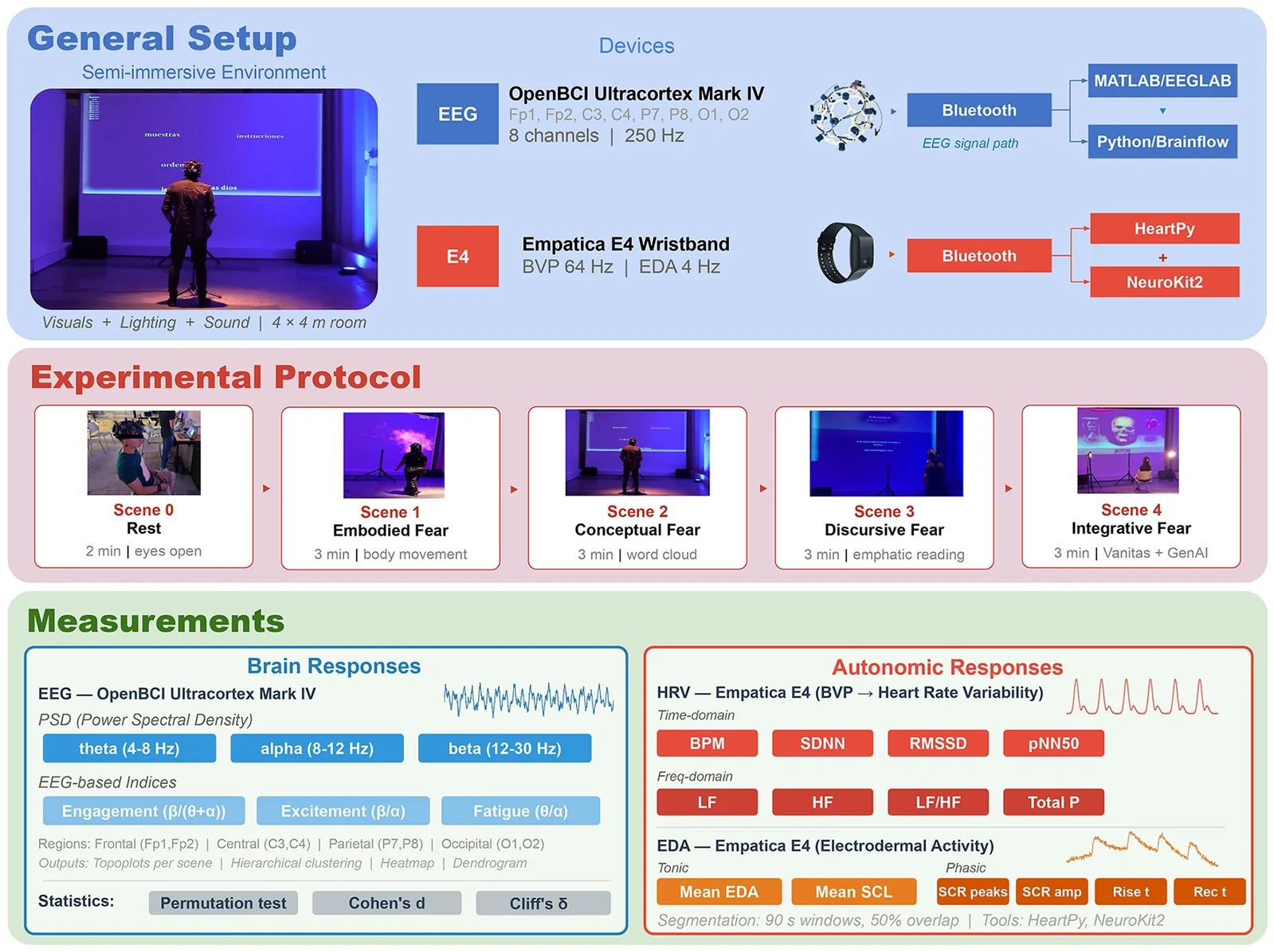
Semi-immersive general setup including visual, auditory, and lighting stimulation, OpenBCI Ultracortex Mark IV EEG, and Empatica E4 wristband for BVP and EDA acquisition. The experimental protocol consisted of baseline and four fear-related scenes: embodied, conceptual, discursive, and integrative. Brain responses were characterized using EEG PSD at θ, α, and β frequency bands and EEG-derived indices (engagement, excitement, and fatigue), while autonomic responses included time- and frequency-domain HRV measures and tonic EDA activity. Statistical analysis included permutation tests, Cohen's d, and Cliff's delta.

More particularly, scene 1 (Embodied Fear) consisted of the physical experience, in which no language is involved, only bodily expression. Participants were exposed to a stressful situation, evoked through images and sounds associated with the experience of fear, which were iteratively co-created by creative artists and the research team to build an immersive environment for closed-loop neurofeedback experience, rather than using standardized affective stimuli. Participants were invited to react physically to the environment, while their movements altered the distance of the projection and generated specific sounds as they moved their limbs, thereby creating a semi-immersive experience. Throughout all scenes, EEG and autonomic signals were recorded to form the dataset analyzed offline in this study, utilizing Unix timestamps for cross-device synchronization. Simultaneously, a parallel stream of these signals was processed online using the pipeline described by Ortiz-Espinoza et al. (2025) to drive the adaptive audiovisuals. At the same time, an algorithm calculated a real-time categorical fear score based on an EEG-based Valence-Arousal-Dominance (VAD) emotion model (Blanco-Ríos et al., 2024), previously trained on the Database for Emotion Analysis using Physiological Signals (DEAP) (Koelstra et al., 2012). Following Bălan et al. (2019), VAD values were projected into a 3D emotional space to determine four fear levels across the hate-overconfident diagonal: (1) no fear [*V*: (7, 9), *A*: (1, 3), *D*: (7, 9)], (2) low fear [*V*: (5, 7), *A*: (3, 5), *D*: (5, 7)], (3) medium fear [*V*: (3, 5), *A*: (5, 7), *D*: (3, 5)], and (4) high fear [*V*: (1, 3), *A*: (7, 9), *D*: (1, 3)]. This score was displayed to participants in real-time and modified the intensity of the environment across scenes, making it more intense or more relaxed depending on whether a higher or lower fear level was detected, respectively. Similarly, the flashing of LED lights accompanied these detected ranges by blinking with greater or lesser intensity.

Scene 2 (Conceptual Fear) consisted of a reflexive exercise in which participants presented a conceptual explanation of their previous experience. On the screen, a cloud of words associated with the emotion of fear was projected. The participants said aloud into a microphone the words that best represented the experience of fear, or proposed alternative words. These suggestions were generated using a recommendation system based on word representations extracted from a predefined corpus. The corpus treats the emotion of fear, comprising contemporary novels, newspaper articles, social media posts, and essays.

Scene 3 (Discursive Fear) involved applying the previous concepts in articulated sentences, going beyond the conceptual sphere of knowledge into the discursive one, where nuances such as meaning and emphasis were taken into account. The screen displayed the text fragments that most closely matched the words selected in Scene 2, based on Jaccard similarity. Each paragraph consisted of two or three sentences. The participant was then asked to read the fragments out loud into the microphone in the most emphatic and comprehensible manner.

Scene 4 (Integrative Fear) ends with a symbolic synthesis of the whole experience, appealing to figurative images that can embody different meanings and interpretations of the same fact. The participants were seated, and a camera recognized their facial features through MediaPipe. The Vanitas painting was projected, and they observed it and explored the emotions it evoked by engaging facially. This was done online through GenAI by leveraging a NVIDIA GeForce RTX 3090 eGPU (24 GB VRAM), explained in greater detail in (Ortiz-Espinoza et al., 2025).

### 2.4 Signal pre-processing

EEG data were pre-processed by first assigning the respective EEG channel positions (Fp1, Fp2, C3, C4, P7, P8, O1, O2). Performing the standarized pre-processing (PREP) pipeline (v0.57) (Bigdely-Shamlo et al., 2015) via MATLAB R2024a (MathWorks, Natick, MA) and *EEGLAB* toolbox (v2024.0) (Delorme and Makeig, 2004), which detrends using a high-pass filter (1 Hz), removes line noise by using multiples of 60 Hz, and re-references the EEG data through a robust average reference, which iteratively identifies bad channels in order to interpolate them using the other channels. Furthermore, a Finite Impulse Response (FIR; v2.8; 4–30 Hz) was applied; later, Artifact Subspace Reconstruction (ASR) algorithm (v2.0) (Mullen et al., 2013) (κ = 20) was implemented, which improves signal quality by correcting movement-related artifacts (Cruz-Garza et al., 2020); finally, an Independent Component Analysis (ICA) was performed to classify and remove the non-brain components, including line noise, eye, heart, and muscle, with ICLabel (v1.6).

Using this EEG pipeline, all windows were retained, as ASR and PREP automatically managed artifact correction and bad channel interpolation, respectively. Non-brain components were identified spatially, spectrally, and temporally using ICLabel; those with ≥70% brain probability were retained, removing a maximum of two non-brain components per participant. Analysis was restricted to complete datasets (calibration plus four scenes). Out of 10 subjects, two were excluded: one due to a corrupted calibration file (S5), and one due to rank-deficiency of 1 during ICA caused by channel interpolation (S1); with an effective sample of eight subjects.

BVP signals were denoised using an adaptive frequency-domain approach based on partial Discrete Fourier Transform (DFT) reconstruction, as presented in (Sadhukhan et al., 2018). For each recording, the raw BVP signal was segmented into 10 s windows with 2% overlap. Low- and high-cut frequencies were estimated from the first signal segment. The low-cut frequency was derived from HR estimation with HeartPy toolkit (v1.2.7) (van Gent et al., 2018) following high frequency noise attenuation with an 8 Hz Butterworth low-pass filter. The high-cut frequency was determined from the signal spectrum as the highest frequency whose magnitude exceeded 15% of the dominant spectral peak above the low-cut threshold. Frequencies outside of the estimated band of interest were then rejected from each segment, and signal reconstruction was performed via inverse DFT. Finally, the preprocessed BVP signal was obtained by concatenating the reconstructed segments, discarding overlapping sections.

EDA signals were preprocessed using a data-driven empirical iterative method adapted from (Gautam et al., 2018). For each recording, the raw signal was iteratively smoothed. At each iteration, local maxima and minima were detected and midpoints between paired extrema were computed. A cubic spline was then fitted through these midpoint values to obtain a smoothed component, which would then undergo the same treatment. This procedure was repeated for up to seven iterations, as suggested in the original methodology, or stopped earlier if insufficient extrema pairs were detected. Finally, the last smoothed component would be substracted from the original EDA signal to obtain a residual to be used for subsequent analysis.

### 2.5 Signal processing

Pre-processed EEG brain signal was processed employing Python (v3.9.0) and *Brainflow* (v5.6.0). Using a frequency analysis based on the Fast Fourier Transform (FFT), a 1-s PSD with a half-second overlap was calculated. Afterwards, the average power for each of the following band ranges was calculated: Theta (θ, 4–8 Hz), alpha (α, 8–12 Hz), and beta (β, 12–30 Hz). Afterwards, the averaged pooled brain activity was calculated for each brain region: Frontal (FP1, FP2), Central (C3, C4), Parietal (P7, P8), and Occipital (O1, O2).

In addition, taking into account the combined average brain activity by region, frequency band ratios were also calculated, considering an average of theta at frontal (Fp1, Fp2) and central region (C3, C4), an average of alpha at parietal region (P7, P8), and an average of beta at frontal region (Fp1, Fp2), according to Kamzanova et al. (2011). The EEG-based indices calculated were: Engagement index (Equation 1a), considering cognitive processing in contrast to a more passive state (Pope et al., 1995); Excitement index (Equation 1b), defined as high alert and attentive (Pfurtscheller and Lopes da Silva, 1999); and Fatigue Index (Equation 1c), with mental weariness and required at sustained attention (Ramírez-Moreno et al., 2021).


$$\begin{array}{ll} \text{Engagement}\left(\theta ,\alpha ,\beta \right) & =\frac{\beta }{\theta +\alpha } \end{array}$$ (1a)

$$\begin{array}{ll} \text{Excitement}\left(\alpha ,\beta \right) & =\beta /\alpha \end{array}$$ (1b)

$$\begin{array}{ll} \text{Fatigue}\left(\theta ,\alpha \right) & =\theta /\alpha \end{array}$$ (1c)

Pre-processed Empatica E4 data (BVP and EDA) were segmented into 90 s windows with 50% overlap. Feature extraction was performed independently for each participant and condition, and features were subsequently concatenated across modalities within aligned windows. Given the limited length of the available recordings, HRV features were obtained using an ultra-short-term analysis approach. Accordingly, a window length of 90 s was selected based on recommendations summarized in Shaffer and Ginsberg (2017). This duration is primarily suitable for estimating metrics that reflect rapid beat-to-beat variability, particularly the root mean square of successive RR interval differences (RMSSD) and HF power. Nevertheless, HRV estimates derived from 90 s segments should not be regarded as interchangeable with those obtained from conventional 5 min recordings, particularly for metrics that require longer observation periods (Shaffer and Ginsberg, 2017; Baek et al., 2015; Munoz et al., 2015; Shaffer et al., 2020). To maintain temporal consistency across modalities and facilitate window-level feature integration, EDA signals were segmented using the same 90 s window length. A 50% overlap produced a 45 s step, allowing the initial, intermediate, and final portions of each condition to be represented while balancing temporal resolution and redundancy. Although no physiologically established overlap percentage exists for HRV or EDA, overlapping windows have been used in continuous cardiovascular and electrodermal monitoring (Hovsepian et al., 2015; Gjoreski et al., 2016; Schmidt et al., 2018; Nath and Thapliyal, 2021). Using the aforementioned pipeline, all windows were retained for analysis. Out of 10 subjects, two were excluded due to corrupted files (S7, S10); with an effective sample of eight subjects.

For BVP, peak detection and inter-beat interval (IBI) series estimation were conducted using the HeartPy toolkit (van Gent et al., 2018). For this, a smoothed version of the signal (resulting from the application of a moving average filter) was used as a reference for peak detection. Detected peaks were then verified and corrected, and the resulting RR intervals were cleaned from outliers using the quotient filter method (Piskorski and Guzik, 2007). From the corrected RR series, heart rate (to be referred to as BPM from here onward) as well as HRV features were obtained for each time window. The computed time-domain HRV metrics included the standard deviation of NN intervals (SDNN), root mean square of successive RR interval differences (RMSSD), and the percentage of successive RR intervals that differed by more than 50 ms (pNN50). Frequency-domain metrics were estimated from the power spectral density of the corrected RR series using Welch's method, with LF power (0.04–0.15 Hz), HF power (0.15–0.40 Hz), and total power computed by numerical integration of the corresponding spectral bands. The LF/HF ratio was subsequently calculated from the estimated LF and HF powers (Shaffer et al., 2014). All HRV metrics were computed exclusively from accepted RR intervals after artifact rejection and outlier correction.

As for EDA, each window was processed using NeuroKit2 (Makowski et al., 2021) to separate the slowly varying tonic component, or skin conductance level (SCL), from the faster phasic component containing skin conductance responses (SCRs). SCR events were automatically identified from the phasic component based on their onset, peak, and half-recovery points. The extracted features comprised mean EDA and mean SCL, representing the average overall and tonic skin conductance within each window, respectively; SCR peak count, defined as the number of detected phasic responses within a window; cumulative SCR amplitude (ΣSCR_*A*_), calculated as the sum of the amplitudes of all detected responses; and mean SCR amplitude (SCR_*A*_), calculated as their average amplitude. For each response, amplitude was measured as the increase in conductance from SCR onset to peak. Mean SCR rise time (SCR*t*_*rise*_) represented the average interval from response onset to peak, whereas mean SCR recovery time (SCR*t*_*rec*_) represented the average interval from the peak to the point at which the response decreased to half of its amplitude. These measures characterize the frequency, magnitude, and temporal dynamics of phasic sympathetic electrodermal activity (Posada-Quintero and Chon, 2020; Boucsein et al., 2012). It is worth noting that the introduction of phasic features was not intended to represent event-related SCRs linked to discrete stimulus onsets, but to characterize non-specific phasic activity throughout each scene. Similar studies have extracted features from continuous EDA signals without assigning responses to specific events (Veeranki et al., 2024; Kumar et al., 2023). Accordingly, these features were interpreted as window-level descriptors of the frequency, magnitude, and temporal morphology of sympathetic fluctuations.

Normalization was done through *z*-score, considering each participant's mean (${\bar{x}}_{R}$) and standard deviation (*s*_*R*_) at their 2-min baseline period. Thus, each non-normalized sample *x* was transformed into a normalized sample *x*′ according to Equation 2.


$$\begin{array}{l} x^{\prime }=\frac{\left(x-{\bar{x}}_{R}\right)}{s_{R}} \end{array}$$ (2)

Afterwards, to obtain an average value for each scene, the median of each brain region-frequency band combination and autonomic features was computed from normalized values, averaged within subjects. This median was used to create both the topoplots using Python (*MNE*, v1.5.1) and the multi-modal normalized heatmap using R (*lattice*, v4.5.1). Median instead of average across subjects was used to capture the sample's overall trend while being robust to outliers (Géron, 2019). Median Absolute Deviation (MAD; Equation 3) was calculated to measure the spread of the data, considering the absolute distance between each subject's data point (*x*_*i*_) and the dataset's median ($\tilde{x}$) (Géron, 2019).


$$\begin{array}{l} \text{MAD}=\text{median}\left(\left|x_{i}-\tilde{x}\right|\right) \end{array}$$ (3)

### 2.6 Statistical analysis

Normalized values were used to detect differences between each fear scene and the baseline, as well as to determine whether normalized responses varied across the four fear scenes, using a two-sided exact permutation test and a Friedman test, respectively. *p*-values were Bonferroni-corrected to control for Type I error inflation due to multiple comparisons (Dunn, 1961).

Cohen's d is a measure of effect size that is standardized for the purpose of comparing the mean values of two groups (Lakens, 2013); effect size could be small (d = 0.2), medium (d = 0.5), or large (d = 0.8) (Cohen, 1988). Paired cohen's d (Rousselet et al., 2017) was calculated through Equation 4, where $\bar{z}$ and *s*_*z*_ represent the mean and standard deviation of the differences between *x*_1_ and *x*_2_, which represent the average value for the fear scene and baseline, respectively.


$$\begin{array}{l} d_{z}=\frac{\bar{z}}{s_{z}};z=x_{1}-x_{2} \end{array}$$ (4)

Additionally, a more robust effect size was also calculated, considering the non-parametric paired Cliff's delta (Cliff, 1996), which estimates the probability that a random value from *x*_1_ is greater than a random value from *x*_2_, minus the reverse (Macbeth et al., 2011). Its ranges consist in negligible (0 <δ <0.14), small (0.14 <δ <0.33), medium (0.33 <δ <0.47), and large (δ > 0.47) (Romano et al., 2006). Cliff's δ was calculated as in Equation 5, where the cardinality symbol # indicates counting, *x*_1_ the scores at the fear scene, *x*_2_ the scores at the baseline, and *N* the number of pairs.


$$\begin{array}{l} \delta =\frac{\#\left(x_{1}>x_{2}\right)-\#\left(x_{1}<x_{2}\right)}{N} \end{array}$$ (5)

Finally, a multi-modal hierarchical clustering was performed on overlapping subjects (*n* = 6; S2, S3, S4, S6, S8, S9) considering their subject-scene values, using Spearman distance and average linkage. Bootstrap resampling for cluster validation (*pvclust*, 1,000 iterations) was performed over these subject-scene values, and clusters were considered statistically supported at Approximately Unbiased (AU) *p*-values ≥95. Cophenetic correlation (*cophenetic*) and Baker's gamma (*cor.dendlist*) evaluated linkage effects across average, complete, ward.D2, and single methods. Finally, the Mantel statistic and quantile significance (*mantel*) tested cross-modal coupling using Spearman distance with 1,000 permutations. Spearman (Equation 6) was selected over Euclidean distance for its robustness to outliers (Boslaugh, 2012), where *r*_*s*_ of two features *X* and *Y* is calculated by their difference between ranks *d*, and the number of subjects *n*.


$$\begin{array}{l} r_{s}\left(X,Y\right)=1-\frac{6\sum_{i=1}^{n}d_{i}^{2}}{n\left(n^{2}-1\right)} \end{array}$$ (6)

## 3 Results

For each fear scene, normalized neural (EEG; Table 1) and autonomic (HRV/EDA; Table 2), each table shows the median (MAD) value of each fear scene. Moreover, Tables 3, 4 show inferential statistics: Two-sided exact permutation test's *p*-value, paired Cohen's *d* and Cliff's δ.

**Table 1:** Median and median absolute deviation (MAD) of baseline-normalized EEG features.

Feature	Baseline	Fear scenes			
		Embodied	Conceptual	Discursive	Integrative
θ_Frontal_	0 (0)	2.09 (2.41)	0.34 (0.74)	**4.12** (4.72)	2.42 (2.51)
α_Frontal_	0 (0)	1.25 (0.96)	0.18 (0.92)	**1.80** (2.05)	0.89 (1.06)
β_Frontal_	0 (0)	3.19 (2.66)	0.08 (1.05)	**11.87** (11.4)	2.80 (2.38)
θ_Central_	0 (0)	3.25 (3.59)	**5.23** (5.88)	3.07 (3.68)	0.81 (1.27)
α_Central_	0 (0)	2.44 (2.92)	**3.44** (4.11)	2.71 (3.25)	0.94 (1.80)
β_Central_	0 (0)	3.28 (3.96)	**6.15** (7.12)	2.53 (3.24)	0.23 (0.71)
θ_Parietal_	0 (0)	–0.12 (0.47)	0.04 (0.46)	**1.89** (2.59)	0.83 (1.31)
α_Parietal_	0 (0)	–0.38 (0.24)	–0.39 (0.13)	**0.40** (1.03)	–0.20 (0.37)
β_Parietal_	0 (0)	0.03 (0.58)	0.54 (0.68)	**1.75** (1.86)	0.56 (0.62)
θ_Occipital_	0 (0)	–0.21 (0.46)	–0.46 (0.42)	**3.81** (4.27)	0.05 (0.60)
α_Occipital_	0 (0)	–0.43 (0.42)	–0.47 (0.38)	**1.62** (2.16)	–0.40 (0.62)
β_Occipital_	0 (0)	0.77 (1.53)	0.05 (1.02)	**3.30** (3.83)	0.07 (0.79)
Fatigue	0 (0)	1.40 (1.01)	1.77 (2.09)	**1.79** (1.37)	1.21 (0.91)
Excitement	0 (0)	**5.94** (4.48)	2.56 (2.89)	2.68 (1.98)	3.60 (2.38)
Engagement	0 (0)	**2.38** (1.88)	0.65 (1.00)	0.94 (0.91)	2.12 (2.41)

The highest median value for each feature is shown in bold.

**Table 2:** Median and median absolute deviation (MAD) of baseline-normalized HRV and EDA features (winsorized at ± 3σ).

Feature	Baseline	Fear scenes			
		Embodied	Conceptual	Discursive	Integrative
BPM	0 (0)	**2.25 (0.75)**	1.73 (1.27)	1.80 (1.08)	–0.21 (0.66)
SDNN	0 (0)	–0.20 (0.70)	–0.64 (0.93)	**0.88 (1.32)**	–0.34 (1.04)
RMSSD	0 (0)	0.27 (1.08)	0.27 (1.48)	**1.47 (0.99)**	0.10 (2.20)
pnn50	0 (0)	0.73 (2.23)	0.18 (2.27)	**2.60 (0.26)**	0.47 (1.46)
LF	**0 (0)**	–0.59 (0.69)	–0.42 (0.40)	–0.09 (0.83)	–0.48 (0.28)
HF	0 (0)	–0.32 (0.44)	–0.29 (0.80)	**0.90 (0.77)**	–0.37 (0.69)
LF/HF	**0 (0)**	–0.73 (0.73)	–0.24 (0.88)	–0.28 (1.38)	–0.56 (0.76)
Total P	0 (0)	–0.51 (0.14)	–0.50 (0.69)	**0.50 (0.70)**	0.00 (0.60)
EDA	0 (0)	–0.24 (1.20)	**0.52 (1.04)**	–0.25 (0.73)	–0.22 (1.15)
SCL	0 (0)	–0.22 (1.18)	**0.53 (1.08)**	–0.14 (0.77)	–0.22 (1.15)
SCR_*P*_	**0 (0)**	–0.06 (0.82)	–0.62 (1.09)	–0.29 (0.76)	–0.07 (1.28)
∑SCR_*A*_	0 (0)	0.89 (1.48)	2.50 (0.50)	**3.00 (0.00)**	1.90 (1.10)
SCR_*A*_	0 (0)	0.40 (1.08)	1.56 (1.19)	**3.00 (0.00)**	1.42 (1.58)
SCR_*t*_rise__	0 (0)	–0.30 (0.42)	0.40 (1.75)	**1.70 (0.95)**	0.15 (0.17)
SCR_*t*_rec__	0 (0)	0.15 (0.70)	0.23 (0.82)	**0.52 (0.86)**	0.18 (0.79)

The highest median value for each feature is shown in bold.

**Table 3:** Inferential statistics on baseline-normalized EEG responses for each brain region and frequency band when compared to baseline.

Feature	Fear scenes (*p*/*d*/δ)			
	Embodied	Conceptual	Discursive	Integrative
θ_Frontal_	0.06/0.69/**0.50**	1.16/0.36/0.25	0.21/0.54/**0.50**	0.10/0.41/**0.50**
α_Frontal_	0.07/0.71/**0.75**	1.30/0.36/0.00	0.19/0.62/**0.50**	0.07/0.50/**0.75**
β_Frontal_	**0.0003**/0.69/**1**	0.78/0.37/0.00	**0.02**/**1.02**/**0.75**	**0.005**/0.54/**0.75**
θ_Central_	0.25/0.55/0.25	0.27/**0.87**/0.25	0.25/0.55/**0.50**	0.28/0.65/**0.50**
α_Central_	0.30/0.55/0.25	0.31/0.79/0.00	0.28/0.56/0.25	0.38/0.63/0.25
β_Central_	0.18/0.65/**0.50**	0.23/0.65/**0.50**	0.30/0.42/0.25	0.64/0.60/0.00
θ_Parietal_	2.24/0.34/0.00	2.17/0.22/0.00	0.30/0.68/0.25	0.26/0.75/0.25
α_Parietal_	4.00/0.04/**–0.75**	**0.02**/**–1.31**/**–0.75**	1.40/0.37/0.00	2.78/0.15/–0.25
β_Parietal_	1.76/0.42/0.25	0.42/0.58/**0.50**	0.10/0.54/**0.5**	0.18/0.68/**0.50**
θ_Occipital_	0.79/0.55/0.00	1.86/0.41/–0.25	0.10/0.53/0.25	0.76/0.56/0.00
α_Occipital_	0.82/0.52/–0.25	1.86/0.41/–0.25	0.28/0.60/0.25	0.8/0.55/–0.25
β_Occipital_	0.42/0.60/0.25	1.67/0.41/0.00	**0.03**/0.63/**0.50**	0.76/0.54/0.00
Fatigue	**0.0003**/0.60/**1**	0.07/0.43/**0.75**	**0.0003**/**0.84**/**1**	**0.0003**/0.68/**1**
Excitement	**0.02**/0.54/**0.75**	0.21/0.38/**0.50**	**0.0003**/0.49/**1**	**0.005**/0.53/**0.75**
Engagement	**0.04**/0.76/**0.75**	0.35/0.47/0.25	**0.005**/0.53/**0.75**	0.10/0.69/**0.50**

Each fear scene was compared with baseline. Two-sided exact permutation test's Bonferroni-corrected *p*-value, paired Cohen's *d* and Cliff's δ. *p* <0.05, |*d*|>0.8 and |δ|>0.47 are in bold.

**Table 4:** Inferential statistics on baseline-normalized HRV and EDA responses when compared to baseline.

Feature	Fear scenes (*p*/*d*/δ)			
	Embodied	Conceptual	Discursive	Integrative
BPM	0.94/0.62/**0.50**	0.35/**0.90**/**0.75**	0.79/0.69/**0.50**	1.00/–0.38/0.00
SDNN	1.00/–0.59/0.00	1.00/–0.23/**–0.50**	1.00/0.45/0.25	1.00/–0.35/–0.25
RMSSD	1.00/0.02/0.00	1.00/0.28/0.00	0.31/**0.94**/**0.75**	1.00/0.00/0.00
pnn50	1.00/0.33/0.25	1.00/0.06/0.00	0.12/**1.20**/**0.75**	1.00/0.12/0.25
LFk	1.00/–0.49/–0.25	0.40/**–0.91**/**–0.50**	1.00/0.29/0.00	1.00/–0.50/**–0.50**
HFk	1.00/–0.48/–0.25	1.00/0.01/–0.25	0.25/**0.98**/**0.50**	1.00/0.15/–0.25
LF/HF	1.00/–0.58/–0.25	1.00/–0.26/0.00	1.00/–0.46/0.00	1.00/–0.57/–0.25
Total Pk	**0.005**/**–1.10**/**–0.75**	1.00/–0.49/–0.25	1.00/0.26/0.25	1.00/–0.02/0.00
EDA	1.00/–0.02/–0.25	0.52/**0.82**/**0.50**	1.00/–0.13/–0.25	1.00/–0.08/–0.09
SCL	1.00/–0.01/0.00	0.52/**0.84**/**0.50**	1.00/–0.10/0.00	1.00/–0.07/–0.09
SCR_*P*_	1.00/0.18/0.00	1.00/–0.15/–0.25	1.00/–0.25/**–0.50**	1.00/–0.41/0.00
∑SCR_*A*_	0.31/**0.96**/0.25	0.10/**1.37**/**0.50**	**0.005**/**2.17**/**0.75**	0.25/**1.06**/0.25
SCR_*A*_	0.63/**0.84**/0.25	0.22/**1.01**/0.25	**0.005**/**2.34**/**0.75**	0.31/**0.93**/0.00
SCR_*t*_rise__	1.00/–0.57/**–0.50**	1.00/0.44/0.00	0.56/0.77/**0.50**	1.00/0.34/**0.50**
SCR_*t*_rec__	1.00/0.25/0.25	1.00/0.37/0.25	1.00/0.50/0.25	0.79/0.67/0.00

Each fear scene was compared with baseline.

Two-sided exact permutation test's Bonferroni-corrected p-value, paired Cohen's d and Cliff's δ. *p* <0.05, |*d*|>0.8 and |δ|>0.47 are in bold.

The normalized EEG topoplots are shown at Figure 2, and their inferential statistics at Figure 4, while the multi-modal *z*-score normalized heatmap with hierarchical clustering is shown at Figure 3.

**Figure 2:**
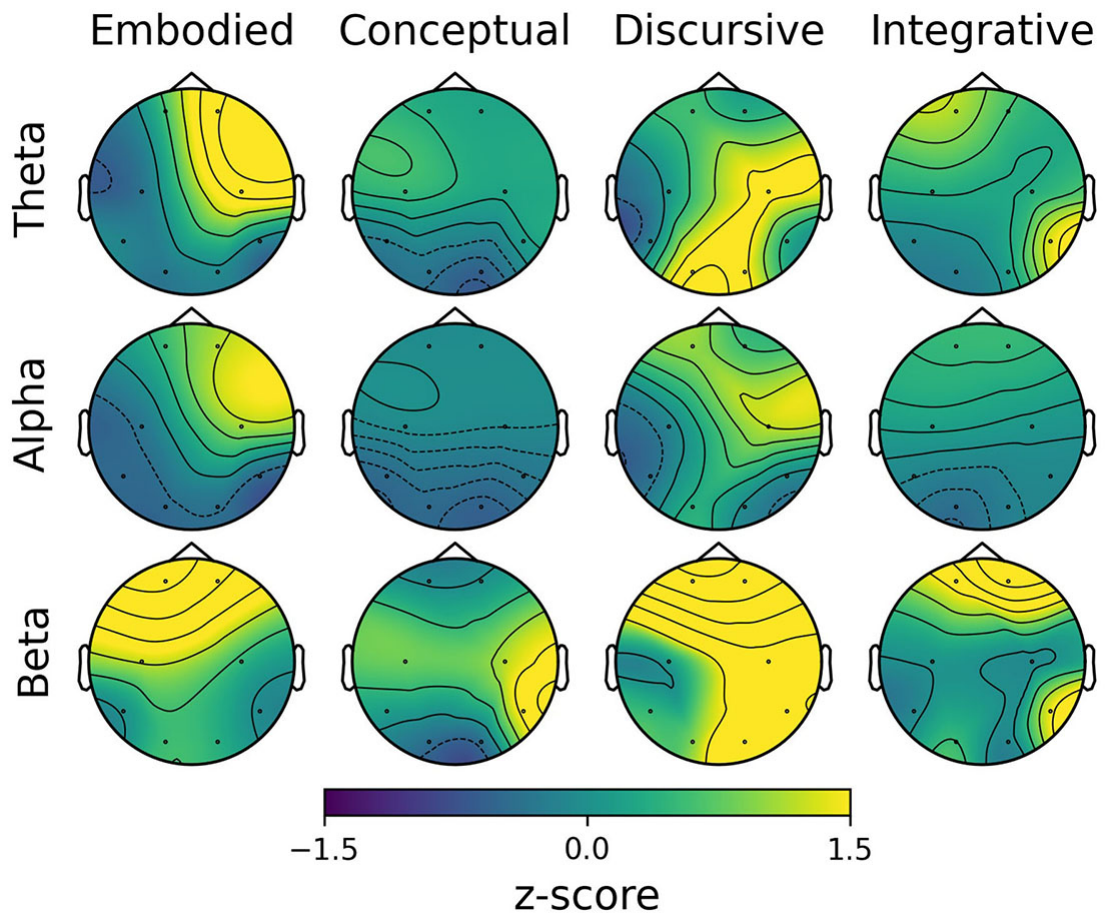
Topoplots of *z*-score baseline-normalized median band power (theta, alpha, beta) for each fear scene (embodied, conceptual, discursive, integrative).

**Figure 3:**
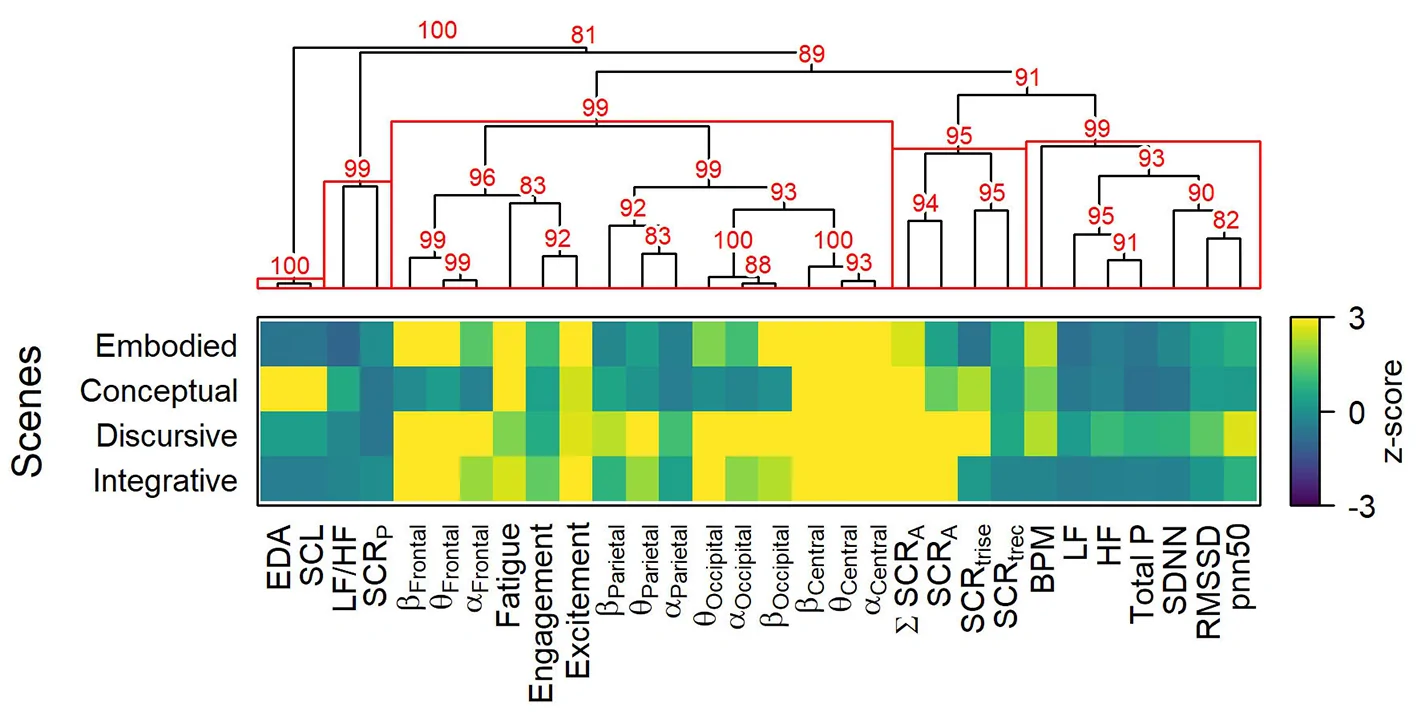
Multi-modal hierarchical cluster of baseline-normalized features (median *z*-score across overlapping subjects). The top dendrogram displays approximately unbiased (AU) *p*-values computed via bootstrap resampling across subjects, with statistically significant clusters (AU ≥95) highlighted in red.

**Figure 4:**
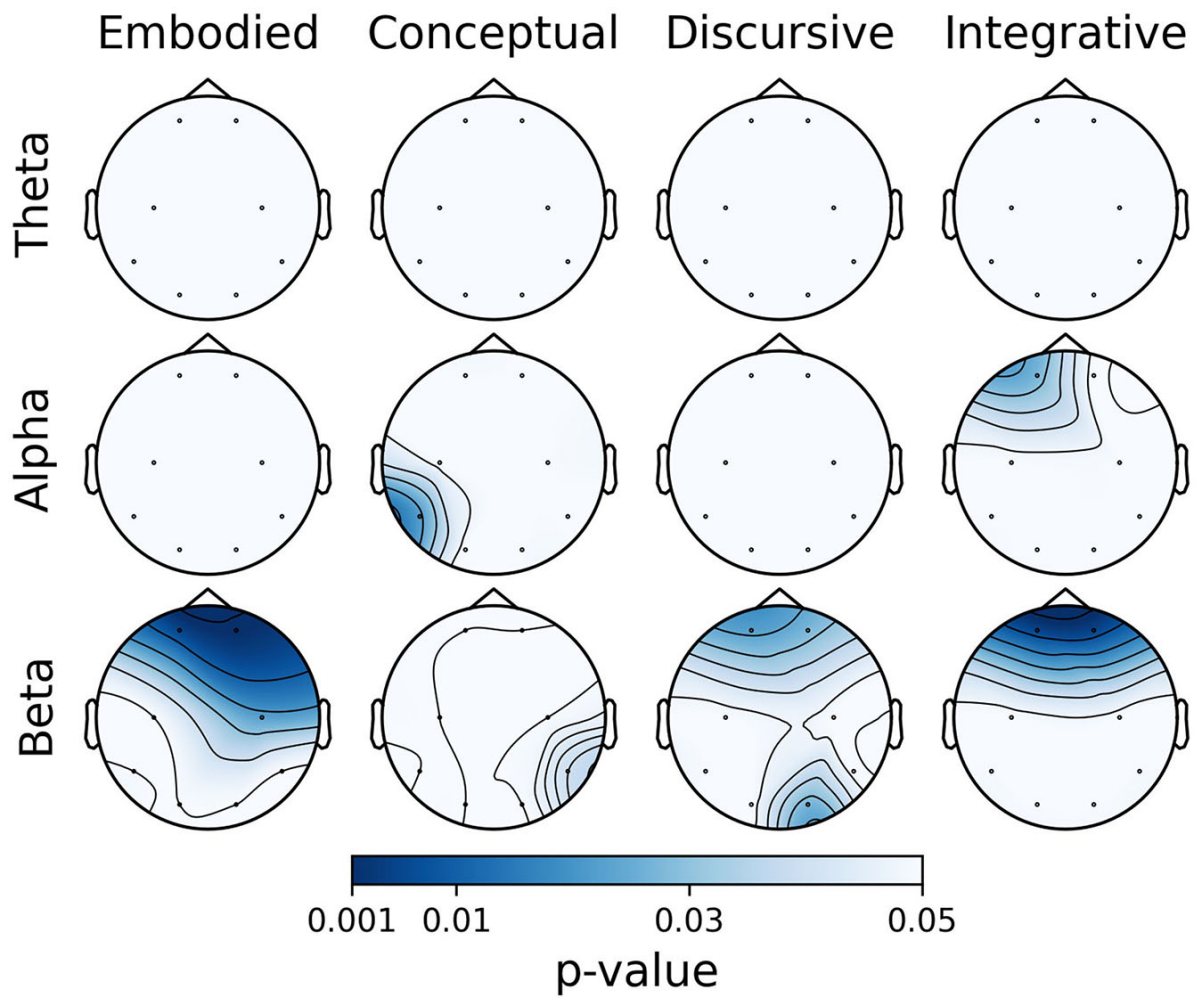
Topoplots of Bonferroni-corrected, exact permutation test *p*-values with baseline-normalized band power (theta, alpha, beta) for each fear scene (embodied, conceptual, discursive, integrative).

### 3.1 Scene-based neurophysiological responses

Considering normalized, *z*-score EEG responses at Table 1 and inferential statistics at Table 3, there are consistent yet varied responses according to the scene in which the participant was situated. Considering the frontal region, on θ, there were no statistically significant *z*-score values, as it was slightly higher during the Conceptual scene (0.34, *p* = 1.16, *d* = 0.36, δ = 0.25), higher at Embodied (2.09, *p* = 0.06, *d* = 0.69, δ = 0.50) and Integrative (2.42, *p* = 0.10, *d* = 0.41, δ = 0.50), and highest at Discursive (4.12, *p* = 0.21, *d* = 0.54, δ = 0.50); α also do not showed statistically significant values, as it was slightly higher for all fear scenes (1.25, 0.18, 1.80, 0.89; *p* = 0.07, *p* = 1.30, *p* = 0.19, *p* = 0.07; *d* = 0.71, *d* = 0.36, *d* = 0.62, *d* = 0.50; δ = 0.75, δ = 0, δ = 0.50, δ = 0.75), with Discursive being the highest (1.80, *p* = 0.19, *d* = 0.62, δ = 0.50); and β had some statistically significant differences at Embodied, Discursive, and Integrative, as it was slightly higher during the Conceptual scene (0.08, *p* = 0.78, *d* = 0.37, δ = 0.00), higher at Integrative (2.80, *p* = 0.005, *d* = 0.54, δ = 0.75) and Embodied (3.19, *p* = 0.0003, *d* = 0.69, δ = 1.00), and highest at Discursive (11.87, *p* = 0.02, *d* = 1.02, δ = 0.75). Moreover, though not statistically significant across frequency bands, *z*-scored θ_Central_ power was slightly higher during the Integrative scene (0.81, *p* = 0.28, *d* = 0.65, δ = 0.50), higher at Discursive (3.07, *p* = 0.25, *d* = 0.55, δ = 0.50), and Embodied (3.25, *p* = 0.25, *d* = 0.55, δ = 0.25), and highest at Conceptual (5.23, *p* = 0.27, *d* = 0.87, δ = 0.25); similarly, α_Central_ was slightly higher during the Integrative scene (0.94, *p* = 0.38, *d* = 0.63, δ = 0.25), higher at Embodied (2.44, *p* = 0.30, *d* = 0.55, δ = 0.25), and Discursive (2.71, *p* = 0.28, *d* = 0.56, δ = 0.25), and highest at Conceptual (3.44, *p* = 0.31, *d* = 0.79, δ = 0.00); likewise, β_Central_ was slightly higher during the Integrative scene (0.23, *p* = 0.64, *d* = 0.60, δ = 0.00), higher at Discursive (2.53, *p* = 0.30, *d* = 0.42, δ = 0.25), and Embodied (3.28, *p* = 0.18, *d* = 0.65, δ = 0.50), and highest at Conceptual (6.15, *p* = 0.23, *d* = 0.65, δ = 0.50).

Among all EEG-derived indices, Fatigue was the most consistent and statistically significant across scenes, having its highest value at Discursive (1.79, *p* = 0.0003, *d* = 0.84, δ = 1.00), following Conceptual (1.77, *p* = 0.07, *d* = 0.43, δ = 0.75), Embodied (1.40, *p* = 0.0003, *d* = 0.60, δ = 1.00), and Integrative (1.21, *p* = 0.0003, *d* = 0.68, δ = 1.00); Excitement index also showed a similar pattern, with the highest value being at Embodied (5.94, *p* = 0.02, *d* = 0.54, δ = 0.75), followed by Integrative (3.60, *p* = 0.005, *d* = 0.53, δ = 0.75), Discursive (2.68, *p* = 0.0003, *d* = 0.49, δ = 1.00), and Conceptual (2.56, *p* = 0.21, *d* = 0.38, δ = 0.50); lastly, Engagement index had mixed responses, as both Embodied and Integrative had higher values (2.38, *p* = 0.04, *d* = 0.76, δ = 0.75; 2.12, *p* = 0.10, *d* = 0.69, δ = 0.50), while Discursive and Conceptual had lower values (0.94, *p* = 0.005, *d* = 0.53, δ = 0.75; 0.65, *p* = 0.35, *d* = 0.47, δ = 0.25).

Lastly, parietal and occipital regions exhibited mixed responses, with non-statistically significant values at θ_Parietal_ (–0.12, 0.04, 1.89, 0.83) and θ_Occipital_ (–0.21, –0.46, 3.81, 0.05), as well as decreasing *z*-score values at α on all scenes except for Discursive, for instance α_Parietal_ (–0.38, –0.39, 0.40, –0.20) and α_Occipital_ (–0.43, –0.47, 1.62, –0.40), with α_Parietal_ at Conceptual being the only statistically significant (–0.39, *p* = 0.02, *d* = −1.31, δ = −0.75); while β had increasing *z*-scores on all scenes, for instance β_Parietal_ (0.03, 0.54, 1.75, 0.56) and β_Occipital_ (0.77, 0.05, 3.30, 0.07), with β_Occipital_ at Discursive being the only statistically significant (3.30, *p* = 0.03, *d* = 0.63, δ = 0.50.

Moreover, channel-level normalized EEG responses are shown with topoplots (Figure 2) of θ, α, and β power for each fear scene (Embodied = E, Conceptual = C, Discursive = D, Integrative = I), as well as statistically significant differences to baseline with topoplots of Bonferroni-corrected exact permutation test's *p*-values (Figure 4). On θ power there were no statistically significant *z*-score values, as the Embodied scene had the highest activation at C4 (E = 2.01, C = 0.24, D = 1.60, I = 0.34) and FP2 (E = 2.32, C = 0.25, D = 0.35, I = 0.59); while the Discursive scene had the highest activation at O1 (E = –0.27, C = –0.33, D = 1.94, I = –0.21) and O2 (E = –0.07, C = –0.53, D = 1.60, I = 0.17), while the Integrative scene had the highest activation at P8 (E = –0.09, C = 0.13, D = 0.37, I = 1.33). Moreover, at the α power, there is a shared neural pattern of desynchronization at the parietal region, particularly, a significant decrease in P7 at Conceptual (E = –0.47, C = –0.43; *p* = 0.022, D = –0.37, I = –0.13) and a descriptively decrease in P8 (E = –0.45, C = –0.31, D = –0.04, I = –0.09); in addition to a synchronization in FP1 at Integrative (E = 0.53, C = –0.03, D = 1.00, I = 0.49; *p* = 0.027) and a descriptively increase in FP2 (E = 1.16, C = –0.03, D = 0.59, I = 0.49) channels, with Embodied and Discursive have non-statistically significant higher values at the C4 channel (E = 1.06, C = –0.09, D = 1.19, I = 0.07) but lower at C3 (E = –0.31, C = –0.06, D = –0.13, I = 0.18). Finally, regarding the β power, there is a significant synchronization pattern at Embodied, Discursive, and Integrative in FP1 (E = 3.13; *p* = 0.005, C = –0.05, D = 7.11; *p* = 0.022, I = –0.32; *p* = 0.005) and FP2 (E = 1.16; *p* = 0.001, C = 0.03, D = 6.87; *p* = 0.027, I = 2.64; *p* = 0.005) channels; in addition to significant increase in P8 power at Conceptual (E = –0.05, C = 1.62; *p* = 0.040, D = 1.73, I = 1.38) and O2 power at Discursive (E = 0.38, C = 0.37, D = 2.58; *p* = 0.027, I = –0.11).

Regarding heart rate and time-domain HRV metrics, none of the comparisons between the fear scenes and baseline remained statistically significant after Bonferroni correction (Table 4). Nevertheless, the descriptive statistics showed that the Discursive scene had the highest median values for SDNN, RMSSD, and pNN50 (Table 2). The largest differences relative to baseline, in terms of median, were observed for RMSSD (1.47, *p* = 0.31, *d* = 0.94, δ = 0.75) and pNN50 (2.60, *p* = 0.12, *d* = 1.20, δ = 0.75), both of which exhibited large effect sizes. BPM was descriptively higher than baseline during the Embodied, Conceptual, and Discursive scenes, with the largest standardized difference observed for the Embodied scene (2.25, *p* = 0.94, *d* = 0.62, δ = 0.50), although non-statistically significant. These findings should therefore be interpreted as descriptive patterns rather than statistically supported differences.

Regarding frequency-domain HRV metrics, only one comparison remained statistically significant after correction. Total power was lower during the Embodied scene (–0.66) than at baseline (*p* = 0.005, *d* = −1.10, δ = −0.75). Among the experimental scenes, Discursive showed the highest median values for HF power (0.90) and total power (0.50); however, neither difference relative to baseline was statistically significant (HF: *p* = 0.25, *d* = 0.98, δ = 0.50; total power: *p* = 1.000, *d* = 0.26, δ = 0.25). The LF/HF ratio was descriptively lower during all experimental scenes than at baseline, although none of these comparisons reached statistical significance. Thus, the frequency-domain results provided statistical support only for lower total power during Embodied, whereas the remaining differences represent descriptive patterns.

Regarding EDA, the tonic measures showed their highest median values during the Conceptual scene. Specifically, EDA appears to have increased from a median of 0 at baseline to 0.52 during Conceptual, although the comparison did not reach statistical significance after correction (*p* = 0.52, *d* = 0.82, δ = 0.50). SCL showed a similar pattern, increasing from a median of 0 at baseline to 0.53 during Conceptual (*p* = 0.52, *d* = 0.84, δ = 0.50). Thus, despite the large effect sizes, these differences should be interpreted as descriptive.

For the phasic EDA metrics, SCR_*P*_ was non-significantly lower during all experimental scenes than at baseline. The largest difference between medians was observed during Conceptual, decreasing from 0 at baseline to –0.62 (*p* = 1.00, *d* = −0.15, δ = −0.25). In contrast, both ∑SCR_*A*_ and SCR_*A*_ showed higher median values during all experimental scenes and reached their maxima during Discursive. The median of ∑SCR_*A*_ increased from 0 at baseline to 3.00 during Discursive, representing a statistically significant difference with large effect sizes (*p* = 0.005, *d* = 2.17, δ = 0.75). Similarly, the median SCR_*A*_ increased from 0 to 3.00 during Discursive (*p* = 0.005, *d* = 2.34, δ = 0.75). The largest differences between medians for the temporal SCR metrics were also observed during Discursive: SCR_*t*_rise__ increased from 0 at baseline to 1.70 (*p* = 0.56, *d* = 0.77, δ = 0.50), whereas SCR_*t*_rec__ increased from 0 to 0.52 (*p* = 1.00, *d* = 0.50, δ = 0.25). However, neither temporal comparison was statistically significant after correction. Overall, statistical support for the phasic EDA responses was limited to the higher ∑SCR_*A*_ and SCR_*A*_ values observed during the Discursive scene.

### 3.2 EEG-autonomic relationships

Further, a multi-modal normalized heatmap with a hierarchical cluster analysis is shown at Figure 3. Average linkage was selected for its highest cophenetic correlation (*c* = 0.70; complete *c* = 0.60; single *c* = 0.58; Ward (*c* = 0.53). Baker's γ indicated strong agreement within linkage families (average-single γ = 0.76; complete-Ward γ = 0.85), but weak agreement across families (γ= 0.19–0.25), indicating that structure is sensitive to linkage method. Additionally, a Mantel test on the Spearman correlation-based EEG and HRV distance matrices found a weak but significant cross-modal association (*r* = 0.082, *p* = 0.045).

Although several statistically significant clusters emerged after bootstrapping (AU≥95), they remained isolated across devices, having no significant higher-level connections (h>0.72, AU <95). Hierarchical clustering yielded five main branches: (1) a significant EDA cluster (EDA and SCL; h = 0.02, AU = 100), (2) a significant cross-modal HRV-EDA cluster (LF/HF and SCR_*P*_; h = 0.45, AU = 99), (3) an EEG cluster, (4) a SCR cluster, and (5) a HRV cluster.

Within the EEG branch, frontal θ and α formed a significant cluster (h = 0.03, AU = 99) before merging significantly with β (h = 0.13, AU = 99). This group later merged significantly (h = 0.41, AU = 96) with a non-significant EEG index cluster, composed of a non-significant Engagement-Excitement group (h = 0.14, AU = 92) and Fatigue (h = 0.38, AU = 83). At h = 0.72, this entire frontal and EEG index cluster merged significantly (AU = 99) with a compound parietal-occipital-central cluster. Here, a non-significant parietal sub-cluster (θ and α at h = 0.15, AU = 83, joined non-significantly by β at h = 0.28, AU = 92) joined a significant occipital-central sub-cluster at h = 0.45 (AU = 99). That sub-cluster was non-significantly formed at h = 0.35 (AU = 93) by combining a non-significant occipital group (α and β at h = 0.02, AU = 88, joined significantly by θ at h = 0.04, AU = 100) with a non-significant central group (θ and α at h = 0.03, AU = 93, joined significantly by β at h = 0.09, AU = 100).

For SCR features, a non-significant sub-cluster of ΣSCR_*A*_ and SCR_*A*_ (h = 0.30, AU = 94) joined a significant SCR_trise_-SCR_trec_ cluster (h = 0.34, AU = 95) to form a significant SCR branch at h = 0.60 (AU = 95). Finally, within HRV features, a non-significant HF-Total P group (h = 0.12, AU = 91) joined LF significantly (h = 0.24, AU = 95). This branch merged non-significantly at h = 0.50 (AU = 93) with a secondary branch formed by a non-significant RMSSD-pNN50 group (h = 0.22, AU = 82) and SDNN (h = 0.34, AU = 90). Both sub-clusters ultimately merged significantly with BPM at h = 0.63 (AU = 99).

## 4 Discussion

Participants exhibited shared neural and autonomic patterns across all fear scenes compared to baseline, identifying potential markers of fear. Additionally, unique responses at each scene also showed contextual modulation. Finally, multi-modal integration was essential for understanding the psychophysiological interplay between neural and autonomic dynamics during these fear scenes.

### 4.1 Neural and autonomic signatures of fear

The most consistent scene-invariant trend was the statistically significant increased activation of β_Frontal_ for all scenes except for the Conceptual scene (Tables 1, 3), also seen at the channel-level considering FP1 and FP2 (Figures 2, 4). Frontal regions are critical on fear-related neural circuity (Åhs et al., 2015; Picó-Pérez et al., 2019; Milad and Quirk, 2012; Rezaei et al., 2025; Kim et al., 2019; DeLaRosa et al., 2014; Apicella et al., 2023; Demaree et al., 2005), as they play a central role in fear and cognitive control (Kim et al., 2019; Demaree et al., 2005), and β in particular has been associated with increased attentional demands (Kim et al., 2019), perceptual processing (Okazaki et al., 2008), action execution (Lamarche and Ogilvie, 1997), and fear-related cognition (Apicella et al., 2023). Moreover, there was a descriptive increase of θ_Central_ and θ_Frontal_ across scenes, which has been related with increased decision-making and planning (DeLaRosa et al., 2014), as well as mental effort (Candela-Leal et al., 2025b; Villafaina et al., 2021b). Furthermore, α_Parietal_ showed a significant decrease at the Discursive scene, as well as descriptive decrease at Embodied and Integrative scenes, this region has been associated with emotional processing (Ugalde-Canitrot et al., 2025; Alemayehu et al., 1995; Yaşgüçlükal et al., 2021) and visual spatial attention (Rihs et al., 2007; Sauseng et al., 2005; Mann et al., 1996; Villafaina et al., 2021a), with its α downregulation linked to immersive virtual environments (Botrel et al., 2025; Magosso et al., 2019). Consequently, given Fatigue (θ/α), Excitement (β/α), and Engagement (β/(θ+α)) indices definition, and that both θ_Central_ and β_Frontal_ increased, as well as that α_Parietal_ decreased, EEG-derived indices significantly increased across all scenes except for the Conceptual scene, and Engagement for the Integrative scene. This was expected as the increased visual and auditory stimulation often increases mental weariness and alertness (Ramírez-Moreno et al., 2021; Pfurtscheller and Lopes da Silva, 1999; Pope et al., 1995; Candela et al., 2026; Romo-De León et al., 2026). Ultimately, the statistically significant modulations of α and β bands in are in line with earlier fear-related processing findings that considered θ and β bands (Rezaei et al., 2025), as well as α, β, and γ bands (Russo et al., 2024).

Regarding autonomic responses (Tables 2, 4), BPM was descriptively higher than baseline during the Embodied, Conceptual, and Discursive scenes, although none of these differences remained statistically significant after correction. This pattern may be compatible with increased autonomic arousal, effort, or threat-related activation during immersive tasks (Stiedl and Hager, 2017; Luong and Holz, 2022; Malińska et al., 2015). This is to mean that the observed behavior cannot be attributed exclusively to fear. The experimental scenes differed from the resting baseline in motor activity, cognitive engagement, verbal demands, and audiovisual stimulation. These factors may independently affect BPM and may also introduce variation or artifacts into wrist-recorded signals (Nelson et al., 2020; Menghini et al., 2019; Aisy Al et al., 2024; Shaffer and Ginsberg, 2017; Luong and Holz, 2022; Malińska et al., 2015; Lebamovski et al., 2025). Accordingly, the observed BPM pattern could be more appropriately interpreted as reflecting the combined demands of each scene rather than a fear-specific fight-or-flight response.

For EDA, the significantly higher ∑SCR_*A*_ and SCR_*A*_ values during the Discursive scene suggest increased phasic sympathetic activity under this condition. This interpretation is consistent with studies reporting larger or more frequent SCRs during fear, stress, and immersive experiences (Ghiasi et al., 2020; Yu et al., 2024; Kreibig et al., 2007; Luong and Holz, 2022). Nevertheless, these window-level metrics represent non-specific phasic activity and cannot be linked to individual fear-related events. Moreover, the remaining EDA features, including the relatively lower SCR_*P*_ values and the higher SCR *t*_rise_ and SCR *t*_rec_ values during some scenes, did not remain statistically significant after correction and should therefore be regarded as exploratory findings. An interesting pattern is that of the relatively higher pNN50 and RMSSD values during the Discursive scene and the generally lower LF/HF ratios, as they may hint parasympathetic predominance (which contradicts the observed phasic behavior) (Thomas et al., 2019; Shaffer and Ginsberg, 2017; Kleiger et al., 2005), however, this should not be interpreted as conclusive evidence given the lack of statistical significance. Moreover, these indices are sensitive to respiratory patterns, which were not monitored or controlled during the experiment (Schipke et al., 1999; Grossman and Taylor, 2007). Verbal activity, movement, task engagement, and the characteristics of the semi-immersive environment may therefore have contributed to the apparently mixed autonomic patterns (Nelson et al., 2020; Menghini et al., 2019; Aisy Al et al., 2024; Shaffer and Ginsberg, 2017; Luong and Holz, 2022; Malińska et al., 2015; Lebamovski et al., 2025). Overall, the findings suggest a complex autonomic response to the scenes, but the present design does not allow the effects of fear to be separated from those of respiration, behavioral demands, sensory stimulation, and measurement-related variation. Further studies incorporating respiratory and movement monitoring, together with appropriately matched active control conditions, are needed to clarify these contributions.

### 4.2 Contextual modulation of fear

Even though there were shared autonomic responses and neural activity across scenes, some had distinct modulations; thus, characterizing participants' responses to each scenario. While permutation tests revealed statistical significant differences between each scene and the baseline, a Friedman test testing for overall differences among the four scenes themselves was non-significant across EEG, HRV, and EDA (*p*>0.05). Inter-scene differences are thus treated as descriptive rather than confirmatory.

Even though the Embodied, Discursive, and Integrative scenes showed statistically significant increases in β_Frontal_, β_FP1_, and β_FP2_, the Conceptual scene did not. Interestingly, the Conceptual was the only scene to exhibit an statistically significant decrease of α_Parietal_, and the channel-level topoplot (Figure 4) revealed a left-lateralized decrease at α_P7_, aligning with previously reported emotional lateralization effects in immersive fear paradigms, which reflect diverse cognitive demands during fear processing (DeLaRosa et al., 2014; El Basbasse et al., 2023). For instance, β_Frontal_ is related to perception (Kim et al., 2019; Okazaki et al., 2008) and action execution of fear (Lamarche and Ogilvie, 1997; Apicella et al., 2023), while α_Parietal_ to visual spatial attention (Rihs et al., 2007; Sauseng et al., 2005; Mann et al., 1996; Villafaina et al., 2021a) and threat salience (Chien et al., 2017; Codispoti et al., 2023; Arana et al., 2022). Moreover, despite similar Fatigue and Excitement indices across scenes, there was reduced Engagement for Conceptual and Discursive scenes when compared to other scenes, and given indices definition, these differences are derived from increased θ_Frontal_ power, related with enhanced cognitive planning processes (DeLaRosa et al., 2014). Another interesting finding was the descriptive increase of β_Parietal_ and β_P8_ activity during Discursive and Integrative fear, as well as the statistically significant increase of β_P8_ during Conceptual fear, which has been associated with conceptual-level fear generalization (and Morey et al., 2020), belief updating (Hein and Herrojo Ruiz, 2022), and memory-level threat processing (Wannemueller et al., 2024); while decreases have been linked to attention to threat and motor preparation (Sperl et al., 2021; Pirazzini et al., 2023); and right-hemispheric predominance has been related to negative emotion processing (El Basbasse et al., 2023; Tomarken et al., 1990; Prete et al., 2015; Ahern and Schwartz, 1979). These results suggest distinct cognitive demands across conditions: Embodied elicited a bottom-up sensory processing and action execution, Conceptual engaged top-down belief updating and threat salience, while Discursive and Integrative scenes involved a blend of both.

On the other hand, the autonomic pattern observed during Discursive fear may suggest context-dependent modulation, as this scene showed some of the largest descriptive differences in HRV and EDA metrics relative to baseline. Although the higher BPM and EDA responses observed during some scenes are compatible with sympathetic activation associated with fear exposure and threat-related arousal (Stiedl and Hager, 2017; Luong and Holz, 2022; Malińska et al., 2015), they cannot be attributed exclusively to fear. Movement, cognitive engagement, verbal demands, and other characteristics of the experimental tasks may independently influence autonomic activity (Nelson et al., 2020; Menghini et al., 2019; Aisy Al et al., 2024). During Discursive fear, ∑SCR_*A*_ and SCR_*A*_ were significantly higher than at baseline, suggesting increased phasic sympathetic activity under this condition. However, the Discursive scene also showed descriptively higher RMSSD, pNN50, and HF power, although these differences were not statistically supported. Considering the nature of the task, this apparently mixed autonomic pattern may partly reflect the verbal and respiratory demands of emphatic speech, as speech production can simultaneously increase sympathetic activation and modify vagally mediated HRV through respiration-related changes during vocalization (Dodo and Hashimoto, 2019). Because HF power, RMSSD, and pNN50 are strongly influenced by respiration (Schipke et al., 1999; Grossman and Taylor, 2007), these descriptive differences may reflect speech-related respiratory modulation rather than parasympathetic activation attributable to fear. Moreover, verbalization itself has been associated with regulatory effects during unpleasant emotional processing, including reduced sympathetic dominance and increased parasympathetic-related activity (Rantanen et al., 2021). Thus, the autonomic pattern observed during Discursive fear may reflect cognitively mediated and context-dependent processing together with the specific behavioral demands of this scene, rather than a purely reflexive threat response. However, the Friedman tests identified no significant differences among the four fear scenes. Therefore, the present results do not provide strong enough statistical support for scene-specific autonomic modulation, and the comparatively pronounced pattern observed during Discursive fear should be regarded as descriptive and hypothesis-generating.

### 4.3 Multi-modal integration

Multi-modal hierarchical clustering with bootstrap validation on overlapping subjects revealed a modality-specific organization rather than broad brain-autonomic coupling. Five main branches aligned with signal type: EDA (AU = 100), SCR (AU = 95), HRV (AU = 99), and EEG, with neural and autonomic branches remaining statistically independent (h>0.72, AU <95). The only validated cross-modal link (HRV-EDA) joined LF/HF with SCR_*P*_ (h = 0.45, AU = 99), consistent with shared parasympathetic/sympathetic influence on autonomic arousal (Shaffer and Ginsberg, 2017; Thomas et al., 2019). Within EEG, the frontal region (θ, α, β) formed a stable cluster (AU = 99), consistent with attentional engagement during sustained threat processing (Rihs et al., 2007; Sauseng et al., 2005); however, deeper merges with parietal-occipital-central structures were non-significant (AU <95). Finally, a weak yet significant global EEG-autonomic Mantel association (*r* = 0.082, *p* = 0.045) indicated shared variance without the cross-modal clustering required to confirm strong brain-body coupling.

Concurrent brain and autonomic responses highlight the value of multi-modal approaches for enabling a more comprehensive characterization of fear-related processing across brain and body domains, providing greater robustness than unimodal analyses (Ramírez-Moreno et al., 2026; Cos et al., 2023) and supporting its potential utility for interactive and immersive learning environments (Bălan et al., 2020a,b; Freeman et al., 2018).

### 4.4 Limitations

Although consistent neural and autonomic responses were observed across fear-related scenes, together with context-dependent modulation of fear neurophysiology measured through EEG and HRV/EDA, some limitations should be acknowledged.

First, the relatively small sample size (*N* = 10), along with the reduced effective sample sizes for inter-scene comparisons (*N* = 8) and multi-modal hierarchical clustering (*N* = 6), limits statistical generalization; however, multi-modal neurophysiological studies that integrate EEG and peripheral signals are commonly constrained to small cohorts due to device synchronization, pre-processing, and experimental complexity (Ramírez-Moreno et al., 2026), reporting sample sizes of 1 (Pineda-Hernández, 2022; Fuentes-García et al., 2022), 10 (Botrel et al., 2025), 11 (Villafaina et al., 2021b), 12 (Romo-De León et al., 2024), 13 (Olivas et al., 2021), and 14 (Ayuso-Moreno et al., 2020; Villafaina et al., 2019; Yu et al., 2024). Consequently, the present exploratory study emphasized effect sizes and overall response patterns, supported by Cohen's d and Cliff's delta, rather than population-level inference. Future larger cohorts studies could validate both shared and scene-specific findings.

While scenes' variations in task demands, movement, and audiovisual stimulation were inherent to the design's ecological validity, they introduce potential confounding factors. Although responses were normalized to the neutral baseline, the absence of non-fear controls means we could not isolate fear from task or environment-driven effects, such as α band downregulation and increased engagement associated with immersive environments (Botrel et al., 2025; Candela et al., 2026), or cumulative carry-over effects from the fixed order constructive design. Nevertheless, ~1-min washout periods were used to explain the next scene, interrupt immersion, and mitigate physiological carry-over, future studies should also employ continuous recordings to quantify if these intervals returned signals to baseline. In addition, respiration was not controlled and may have influenced autonomic measures such as HF power, RMSSD, and pNN50 (Schipke et al., 1999; Grossman and Taylor, 2007). Moreover, given that the audiovisual environment was adaptively modulated using real-time EEG-derived VAD predictions (Ortiz-Espinoza et al., 2025; Blanco-Ríos et al., 2024), EEG served as a control signal and outcome measure. Hence, the observed EEG differences should be interpreted within the closed-loop paradigm rather than only stimulus-driven effects. Accordingly, while not pilot-testing Scene 1 stimuli for fear induction limits inter-studies direct comparisons, it aligns with the paradigm's ecological focus on adaptive, emotion-modulated interactions.

Besides this, the autonomic findings should not be interpreted as responses exclusively attributable to fear. The resting baseline and experimental scenes were not matched for motor activity, cognitive engagement or audiovisual stimulation, and these factors were not experimentally controlled or managed during data analysis. Movement, in particular, may introduce artifacts into wrist-recorded data, further complicating the distinction between physiological activation and measurement-related variation (Shaffer and Ginsberg, 2017; Boucsein et al., 2012). Consequently, comparisons with baseline should be understood as reflecting the combined autonomic response to each scene rather than a fear-specific effect. Future studies may incorporate respiratory and movement monitoring and include active control conditions matched for the principal motor, cognitive, verbal and sensory characteristics of each scene. Additionally, they may incorporate motion artifact rejection using accelerometry data as a reference signal (e.g., adaptive filtering).

## 5 Conclusions

The present exploratory study identified neural and autonomic signatures of fear during concurrent EEG and peripheral recordings in a semi-immersive environment across multidimensional fear-related scenarios, including Embodied, Conceptual, Discursive, and Integrative.

Shared fear-related responses included frontal and parietal brain activity, increased EEG-derived indices, and elevated BPM and SCR_*A*_ measures, while context-dependent modulation was reflected by scene-specific brain lateralization patterns, with increased β_Frontal_ activity during Embodied fear and decreased α_Parietal_ activity during Conceptual fear, consistent with bottom-up and top-down processing, respectively. Similarly, Discursive fear showed mixed autonomic responses with speech-related respiratory modulation and cognitively mediated rather than reflexive threat processing.

Moreover, multi-modal clustering revealed that fear responses are mainly organized within, rather than across, neural and autonomic systems: features formed robust modality-specific clusters, and while a global EEG-autonomic Mantel association was weak but significant, the only validated cross-modal cluster linked HRV (LF/HF) and EDA (SCR_*P*_). Despite sample size limitations, these findings highlight the value of multi-modal immersive paradigms for capturing distinct and shared dimensions of fear physiology.

## Data Availability

The datasets presented in this study can be found in online repositories. Data is available in FigShare: https://figshare.com/projects/Cartograf_as_Ling_sticas_del_Miedo/203616 Code is available in GitHub: https://github.com/miltoncandela/neurohumanities-lab.

## References

[B1] Åhs F. Kragel P. A. Zielinski D. J. Brady R. LaBar K. S. (2015). Medial prefrontal pathways for the contextual regulation of extinguished fear in humans. NeuroImage 122, 262–271. doi: 10.1016/j.neuroimage.2015.07.051 26220745 PMC4618170

[B2] Ahern G. L. Schwartz G. E. (1979). Differential lateralization for positive versus negative emotion. Neuropsychologia 17, 693–698. doi: 10.1016/0028-3932(79)90045-9 522984

[B3] Aisy Al F. Osmalina Nur R. Khusnul A. Sayyidul Istighfar I. Rifai C. (2024). Measurement of mental workload using heart rate variability and electrodermal activity. IEEE Access 12. doi: 10.1109/ACCESS.2024.3521649

[B4] Alemayehu S. Bergey G. K. Barry E. Krumholz A. Wolf A. Fleming C. P. . (1995). Panic attacks as ictal manifestations of parietal lobe seizures. Epilepsia 36, 824–830. doi: 10.1111/j.1528-1157.1995.tb01621.x 7635102

[B5] Apicella A. Barbato S. Barradas Chacón L. A. D'Errico G. De Paolis L. T. Maffei L. . (2023). Electroencephalography correlates of fear of heights in a virtual reality environment. Acta IMEKO 12, 1–7. doi: 10.21014/actaimeko.v12i2.1457

[B6] Arana L. Melcón M. Kessel D. Hoyos S. Albert J. Carretié L. . (2022). Suppressionof alpha-band power underlies exogenous attention to emotional distractors. Psychophysiology 59:e14051. doi: 10.1111/psyp.14051 35318692 PMC9540775

[B7] Ayuso-Moreno R. Fuentes-García J. P. Collado-Mateo D. Villafaina S. (2020). Heart rate variability and pre-competitive anxiety according to the demanding level of the match in female soccer athletes. Physiol. Behav. 222:112926. doi: 10.1016/j.physbeh.2020.112926 32407830

[B8] Baek H. J. Cho C.-H. Cho J. Woo J.-M. (2015). Reliability of ultra-short-term analysis as a surrogate of standard 5-min analysis of heart rate variability. Telemed. J. E Health 21, 404–414. doi: 10.1089/tmj.2014.0104 25807067

[B9] Bălan O. Moise G. Moldoveanu A. Leordeanu M. Moldoveanu F. (2019). Fear level classification based on emotional dimensions and machine learning techniques. Sensors Cham. 19:1738. doi: 10.3390/s19071738 30978980 PMC6479627

[B10] Bălan O. Moise G. Moldoveanu A. Leordeanu M. Moldoveanu F. (2020a). An investigation of various machine and deep learning techniques applied in automatic fear level detection and acrophobia virtual therapy. Sensors 20:496. doi: 10.3390/s20020496 31952289 PMC7013944

[B11] Bălan O. Moise G. Moldoveanu A. Moldoveanu F. Leordeanu M. (2020b). Classifying the Levels of Fear by Means of Machine Learning Techniques and VR in a Holonic-Based System for Treating Phobias. Experiments and Results (Cham: Springer International Publishing), 357–372. doi: 10.1007/978-3-030-49698-2_24

[B12] Battaglia S. Orsolini S. Borgomaneri S. Barbieri R. Diciotti S. di Pellegrino G. (2022). Characterizing cardiac autonomic dynamics of fear learning in humans. Psychophysiology 59:e14122. doi: 10.1111/psyp.14122 35671393 PMC9787647

[B13] Battaglia S. Thayer J. F. (2022). Functional interplay between central and autonomic nervous systems in human fear conditioning. Trends Neurosci. 45, 504–506. doi: 10.1016/j.tins.2022.04.003 35577621

[B14] Beachaine T. (2001). Vagal tone, development, and gray's motivational theory: toward an integrated model of autonomic nervous system functioning in psychopathology. Dev. Psychopathol. 13, 183–214. doi: 10.1017/S0954579401002012 11393643

[B15] Benedek M. Schickel R. J. Jauk E. Fink A. Neubauer A. C. (2014). Alpha power increases in right parietal cortex reflects focused internal attention. Neuropsychologia 56:393–400. doi: 10.1016/j.neuropsychologia.2014.02.010 24561034 PMC3989020

[B16] Berger H. (1929). Über das elektrenkephalogramm des menschen. Archiv für Psychiatrie und Nervenkrankheiten 87, 527–570. doi: 10.1007/BF01797193

[B17] Berntson G. G. Norman G. J. Hawkley L. C. Cacioppo J. T. (2008). Cardiac autonomic balance versus cardiac regulatory capacity. Psychophysiology 45, 643–652. doi: 10.1111/j.1469-8986.2008.00652.x 18282204 PMC3767155

[B18] Bigdely-Shamlo N. Mullen T. Kothe C. Su K.-M. Robbins K. A. (2015). The PREP pipeline: standardized preprocessing for large-scale EEG analysis. Front. Neuroinform. 9:16. doi: 10.3389/fninf.2015.00016 26150785 PMC4471356

[B19] Blanco-Ríos M. A. Candela-Leal M. O. Orozco-Romo C. Remis-Serna P. Vélez-Saboyá C. S. Lozoya-Santos J. d. J. . (2024). Real-time EEG-based emotion recognition for neurohumanities: perspectives from principal component analysis and tree-based algorithms. Front. Hum. Neurosci. 18:1319574. doi: 10.3389/fnhum.2024.1319574 38545515 PMC10965560

[B20] Boslaugh S. (2012). Statistics in a Nutshell, 2nd Edn. Sebastopol, CA: O'Reilly Media.

[B21] Botrel L. Kreilinger A. Müller M. Pfeiffer M. Scheu V. Vowinkel N. . (2025). The influence of time and visualization on neurofeedback-guided parietal alpha downregulation and sense of presence in virtual reality. Front. Neurosci. 19:1476264. doi: 10.3389/fnins.2025.1476264 40012677 PMC11863144

[B22] Boucsein W. Fowles D. C. Grimnes S. Ben-Shakhar G. Roth W. T. Dawson M. E. . (2012). Publication recommendations for electrodermal measurements. Psychophysiology 49, 1017–1034. doi: 10.1111/j.1469-8986.2012.01384.x 22680988

[B23] Byeon J. Moon J. Y. Je S. R. Park S. H. Kim J. W. (2023). Quantitative electroencephalographic biomarker of pharmacological treatment response in patients with anxiety disorder: a retrospective study. Sci. Rep. 13:3802. doi: 10.1038/s41598-023-30994-1 36882629 PMC9992385

[B24] Candela M. O. Wong-Cantú C. G. Veléz-Saboyá C. S. Félix-Herrán L. C. Tudon-Martínez J. C. Lozoya-Santos J. J. . (2026). “Toward emotionally adaptive learning spaces: brain-body engagement in immersive dance improvisation,” in 2026 IEEE global engineering education conference (EDUCON) (Cairo: IEEE), 1–5. doi: 10.1109/EDUCON67543.2026.11574191

[B25] Candela-Leal M. O. Alanis-Espinosa M. Murrieta-González J. Lozoya-Santos J. d.-J. Ramírez-Moreno M. A. (2025a). Neural signatures of stem learning and interest in youth. Acta Psychol. 255:104949. doi: 10.1016/j.actpsy.2025.104949 40168892

[B26] Candela-Leal M. O. Marrufo-Franco L. A. Ruiz-de-la Fuente B. H. Cruz-Gómez C. F. Ramírez-Moreno M. A. (2026). Closed-Loop Haptic Neurofeedback BCI for Real-Time Student Attention Regulation (Monterrey: Springer Nature Switzerland), 261–269. doi: 10.1007/978-3-032-13729-6_28

[B27] Candela-Leal M. O. Ramirez-Moreno M. A. Lozoya-Santos J. D.-J. (2025b). Task resolution time estimation through cognitive load: an EEG study of chess players. Proc. Annu. Meet. Cogn. Sci. Soc. 47, 2827–2834. Available on line at: https://escholarship.org/uc/item/6qh4q558 (Accessed February 04, 2026).

[B28] Cannon W. B. (1915). Bodily Changes in Pain, Hunger, Fear and Rage: An Account of Recent Researches into the Function of Emotional Excitement. New York, NY: D. Appleton and Company. doi: 10.1037/10013-000

[B29] Castegnetti G. Tzovara A. Staib M. Paulus P. C. Hofer N. Bach D. R. (2016). Modeling fear-conditioned bradycardia in humans. Psychophysiology 53, 930–939. doi: 10.1111/psyp.12637 26950648 PMC4869680

[B30] Chien J. Colloca L. Korzeniewska A. Cheng J. Campbell C. Hillis A. . (2017). Oscillatory EEG activity induced by conditioning stimuli during fear conditioning reflects salience and valence of these stimuli more than expectancy. Neuroscience 346, 81–93. doi: 10.1016/j.neuroscience.2016.12.047 28077278 PMC5426483

[B31] Chirico A. Gaggioli A. (2019). When virtual feels real: comparing emotional responses and presence in virtual and natural environments. Cyberpsychol. Behav. Soc. Netw. 22, 220–226. doi: 10.1089/cyber.2018.0393 30730222

[B32] Christopoulos G. I. Uy M. A. Yap W. J. (2019). The body and the brain: measuring skin conductance responses to understand the emotional experience. Organ. Res. Methods 22, 394–420. doi: 10.1177/1094428116681073

[B33] Cliff N. (1996). Ordinal Methods for Behavioral Data Analysis. Mahwah, NJ: Routledge.

[B34] Codispoti M. De Cesarei A. Ferrari V. (2023). Alpha-band oscillations and emotion: a review of studies on picture perception. Psychophysiology 60:e14438. doi: 10.1111/psyp.14438 37724827

[B35] Cohen J. (1988). Statistical Power Analysis for the Behavioral Sciences, 2nd Edn. New York, NY: Routledge Member of the Taylor and Francis Group.

[B36] Cooper N. Milella F. Pinto C. Cant I. White M. Meyer G. (2018). The effects of substitute multisensory feedback on task performance and the sense of presence in a virtual reality environment. PLoS ONE 13:e0191846. doi: 10.1371/journal.pone.0191846 29390023 PMC5794113

[B37] Cos C.-A. Lambert A. Soni A. Jeridi H. Thieulin C. Jaouadi A. (2023). Enhancing mental fatigue detection through physiological signals and machine learning using contextual insights and efficient modelling. J. Sens. Actuator Netw. 12:77. doi: 10.3390/jsan12060077

[B38] Cruz-Garza J. G. Sujatha Ravindran A. Kopteva A. E. Rivera Garza C. Contreras-Vidal J. L. (2020). Characterization of the stages of creative writing with mobile EEG using generalized partial directed coherence. Front. Hum. Neurosci. 14:577651. doi: 10.3389/fnhum.2020.577651 33424562 PMC7793781

[B39] DeLaRosa B. L. Spence J. S. Shakal S. K. Motes M. A. Calley C. S. Calley V. I. . (2014). Electrophysiological spatiotemporal dynamics during implicit visual threat processing. Brain Cogn. 91, 54–61. doi: 10.1016/j.bandc.2014.08.003 25222294

[B40] Delorme A. Makeig S. (2004). EEGLAB: an open source toolbox for analysis of single-trial EEG dynamics including independent component analysis. J. Neurosci. Methods 134, 9–21. doi: 10.1016/j.jneumeth.2003.10.009 15102499

[B41] Demaree H. A. Everhart D. E. Youngstrom E. A. Harrison D. W. (2005). Brain lateralization of emotional processing: historical roots and a future incorporating “dominance”. Behav. Cogn. Neurosci. Rev. 4, 3–20. doi: 10.1177/1534582305276837 15886400

[B42] Dennis J. K. (2020). The kantian effect: reconceiving the integration of knowledge in interdisciplinary theory. J. Interdiscip. Sci. 4, 1–14. Available online at: https://eric.ed.gov/?id=ED608667 (Accessed February 04, 2026).

[B43] Diemer J. Alpers G. W. Peperkorn H. M. Shiban Y. MÃ¼hlberger A. (2015). The impact of perception and presence on emotional reactions: a review of research in virtual reality. Front. Psychol. 6:26. doi: 10.3389/fpsyg.2015.00026 25688218 PMC4311610

[B44] Diemer J. Lohkamp N. Mühlberger A. Zwanzger P. (2016). Fear and physiological arousal during a virtual height challenge–effects in patients with acrophobia and healthy controls. J. Anxiety Disord. 37, 30–39. doi: 10.1016/j.janxdis.2015.10.007 26600469

[B45] Ding D. Neerincx M. A. Brinkman W.-P. (2020). The effect of an adaptive simulated inner voice on user's eye-gaze behaviour, ownership perception and plausibility judgement in virtual reality. Interact. Comput. 32, 510–523. doi: 10.1093/iwcomp/iwab008

[B46] Dodo N. Hashimoto R. (2019). Autonomic nervous system activity during a speech task. Front. Neurosci. 13:406. doi: 10.3389/fnins.2019.00406 31139041 PMC6518952

[B47] Dunn O. J. (1961). Multiple comparisons among means. J. Am. Stat. Assoc. 56, 52–64. doi: 10.1080/01621459.1961.10482090

[B48] El Basbasse Y. Packheiser J. Peterburs J. Maymon C. Güntürkün O. Grimshaw G. . (2023). Walk the plank! using mobile electroencephalography to investigate emotional lateralization of immersive fear in virtual reality. R. Soc. Open Sci. 10:221239. doi: 10.1098/rsos.221239 37266038 PMC10230188

[B49] Freeman D. Haselton P. Freeman J. Spanlang B. Kishore S. Albery E. . (2018). Automated psychological therapy using immersive virtual reality for treatment of fear of heights: a single-blind, parallel-group, randomised controlled trial. Lancet Psychiatry 5, 625–632. doi: 10.1016/S2215-0366(18)30226-8 30007519 PMC6063994

[B50] Fuentes-García J. P. Leon-Llamas J. L. Gusi N. Villafaina S. (2022). Are there neurophysiological differences behind the play of different chess modalities?: An international grandmaster case study. Physiol. Behav. 255:113918. doi: 10.1016/j.physbeh.2022.113918 35858672

[B51] Gautam A. Simoes-Capela N. Schiavone G. Acharyya A. de Raedt W. Van Hoof C. (2018). “A data driven empirical iterative algorithm for gsr signal pre-processing,” in 2018 26th European signal processing conference (EUSIPCO) (Rome), 1162–1166. doi: 10.23919/EUSIPCO.2018.8553191

[B52] Géron A. (2019). Hands-On Machine Learning With Scikit-Learn and TensorFlow, 2nd Edn. Sebastopol, CA: O'Reilly.

[B53] Ghiasi S. Greco A. Barbieri R. Scilingo E. Valenza G. (2020). Assessing autonomic function from electrodermal activity and heart rate variability during cold-pressor test and emotional challenge. Sci. Rep. 10:5406. doi: 10.1038/s41598-020-62225-2 32214158 PMC7096472

[B54] Gjoreski M. Gjoreski H. Luštrek M. Gams M. (2016). “Continuous stress detection using a wrist device: in laboratory and real life,” in UbiComp '16 (New York, NY: Association for Computing Machinery), 1185–1193. doi: 10.1145/2968219.2968306

[B55] Go C. T. T. H. Leis E. G. Quiambao M. J. D. Samonte M. J. C. Fuentes G. S. Pascua C. A. (2020). “Think+: using virtual reality therapy game mobile application for treating phobia,” in Proceedings of the 6th international conference on frontiers of educational technologies (Tokyo), 130–134. doi: 10.1145/3404709.3404742

[B56] Gromer D. Madeira O. Gast P. Nehfischer M. Jost M. Müller M. . (2018). Height simulation in a virtual reality cave system: validity of fear responses and effects of an immersion manipulation. Front. Hum. Neurosci. 12:372. doi: 10.3389/fnhum.2018.00372 30319376 PMC6167601

[B57] Grossman P. Taylor E. W. (2007). Toward understanding respiratory sinus arrhythmia: relations to cardiac vagal tone, evolution and biobehavioral functions. Biol. Psychol. 74, 263–285. doi: 10.1016/j.biopsycho.2005.11.014 17081672

[B58] Hein T. P. Herrojo Ruiz M. (2022). State anxiety alters the neural oscillatory correlates of predictions and prediction errors during reward-based learning. NeuroImage 249:118895. doi: 10.1016/j.neuroimage.2022.118895 35017125

[B59] Higuera-Trujillo J. L. López-Tarruella Maldonado J. Llinares Millán C. (2017). Psychological and physiological human responses to simulated and real environments: a comparison between photographs, 360° panoramas, and virtual reality. Appl. Ergon. 65, 398–409. doi: 10.1016/j.apergo.2017.05.006 28601190

[B60] Honegger F. Feng Y. Rauterberg M. (2021). Multimodality for passive experience: effects of visual, auditory, vibration and draught stimuli on sense of presence. J. Univers. Comput. Sci. 27, 582–608. doi: 10.3897/jucs.68384

[B61] Hovsepian K. al'Absi M. Ertin E. Kamarck T. Nakajima M. Kumar S. (2015). “cStress: towards a gold standard for continuous stress assessment in the mobile environment,” in UbiComp '15: proceedings of the 2015 ACM international joint conference on pervasive and ubiquitous computing (New York, NY: Association for Computing Machinery), 493–504. doi: 10.1145/2750858.2807526 26543926 PMC4631393

[B62] Jensen O. (2002). Oscillations in the alpha band (9-12 hz) increase with memory load during retention in a short-term memory task. Cerebral Cortex 12, 877–882. doi: 10.1093/cercor/12.8.877 12122036

[B63] Kamzanova A. T. Matthews G. Kustubayeva A. M. Jakupov S. M. (2011). EEG indices to time-on-task effects and to a workload manipulation (cueing). World Acad. Sci. Eng. Technol. 80, 19–22. doi: 10.5281/zenodo.1071802

[B64] Kim M. Cheon S. Kang Y. (2019). Use of electroencephalography (EEG) for the analysis of emotional perception and fear to nightscapes. Sustainability 11:233. doi: 10.3390/su11010233

[B65] Kisker J. Lange L. Flinkenflügel K. Kaup M. Labersweiler N. Tetenborg F. . (2021). Authentic fear responses in virtual reality: a mobile EEG study on affective, behavioral and electrophysiological correlates of fear. Front. Virtual Real. 2:716318. doi: 10.3389/frvir.2021.716318

[B66] Kleiger R. E. Stein P. K. Bigger J. T Jr. (2005). Heart rate variability: measurement and clinical utility. Ann. Noninvasive Electrocardiol. 10, 88–101. doi: 10.1111/j.1542-474X.2005.10101.x 15649244 PMC6932537

[B67] Klimesch W. Doppelmayr M. Schwaiger J. Auinger P. Winkler T. (1999). ‘paradoxical' alpha synchronization in a memory task. Cogn. Brain Res. 7, 493–501. doi: 10.1016/S0926-6410(98)00056-1 10076094

[B68] Koelstra S. Muhl C. Soleymani M. Lee J.-S. Yazdani A. Ebrahimi T. . (2012). Deap: a database for emotion analysis; using physiological signals. IEEE Trans. Affect. Comput. 3, 18–31. doi: 10.1109/T-AFFC.2011.15

[B69] Kreibig S. D. Wilhelm F. Roth W. Gross J. (2007). Cardiovascular, electrodermal, and respiratory response patterns to fear- and sadness-inducing films. Psychophysiology 44, 787–806. doi: 10.1111/j.1469-8986.2007.00550.x 17598878

[B70] Kropotov J. D. (2009). Quantitative EEG, Event-Related Potentials and Neurotherapy. San Diego, CA: Elsevier.

[B71] Kumar S. P. Govarthan P. K. Ganapathy N. Fredo Ar J. (2023). A comparative analysis of EDA decomposition methods for improved emotion recognition. J. Mech. Med. Biol. 22:2340043 doi: 10.1142/S0219519423400432

[B72] Kuplen M. (2023). Kant's Aesthetic Cognitivism. London: Bloomsbury Academic. doi: 10.5040/9781350289543

[B73] Lakens D. (2013). Calculating and reporting effect sizes to facilitate cumulative science: a practical primer for *t*-tests and ANOVAs. Front. Psychol. 4:863. doi: 10.3389/fpsyg.2013.00863 24324449 PMC3840331

[B74] Lamarche C. H. Ogilvie R. D. (1997). Electrophysiological changes during the sleep onset period of psychophysiological insomniacs. psychiatric insomniacs, and normal sleepers. Sleep 20, 726–733. doi: 10.1093/sleep/20.9.726 9406324

[B75] Lebamovski P. Gospodinova E. Georgieva-Tsaneva G. (2025). “Effects of virtual and mixed reality environments on heart rate dynamics during high-intensity gaming,” in 2025 9th international symposium on multidisciplinary studies and innovative technologies (ISMSIT) (Ankara), 1–5. doi: 10.1109/ISMSIT67332.2025.11267828

[B76] Levy M. N. (1971). Sympathetic-parasympathetic interactions in the heart. Circ. Res. 29, 437–445. doi: 10.1161/01.RES.29.5.437 4330524

[B77] Loewi O. (1921). Über humorale Übertragbarkeit der herznervenwirkung. Pflügers Archiv für die gesamte Physiologie des Menschen und der Tiere 189, 239–242. German. doi: 10.1007/BF01738910

[B78] Luck S. J. (2014). An Introduction to the Event-Related Potential Technique. A Bradford Book, 2nd Edn. Cambridge, MA: Bradford Books.

[B79] Luong T. Holz C. (2022). Characterizing physiological responses to fear, frustration, and insight in virtual reality. IEEE Trans. Vis. Comput. Graph. 28, 3917–3927. doi: 10.1109/TVCG.2022.3203113 36048988

[B80] Macbeth G. Razumiejczyk E. Ledesma R. D. (2011). Cliff's delta calculator: a non-parametric effect size program for two groups of observations. Universitas Psychologica 10, 545–555. doi: 10.11144/Javeriana.upsy10-2.cdcp

[B81] Maggioni E. Cobden R. Obrist M. (2019). Owidgets: a toolkit to enable smell-based experience design. Int. J. Hum. Comput. Stud. 130, 248–260. doi: 10.1016/j.ijhcs.2019.06.014

[B82] Magosso E. De Crescenzio F. Ricci G. Piastra S. Ursino M. (2019). EEG alpha power is modulated by attentional changes during cognitive tasks and virtual reality immersion. Comput. Intell. Neurosci. 2019, 1–18. doi: 10.1155/2019/7051079 31341468 PMC6614966

[B83] Makowski D. Pham T. Lau Z. J. Brammer J. C. Lespinasse F. Pham H. . (2021). NeuroKit2: a python toolbox for neurophysiological signal processing. Behav. Res. Methods 53, 1689–1696. doi: 10.3758/s13428-020-01516-y 33528817

[B84] Malińska M. Zuzewicz K. Bugajska J. Grabowski A. (2015). Heart rate variability (HRV) during virtual reality immersion. Int. J. Occup. Saf. Ergon. 21, 47–54. doi: 10.1080/10803548.2015.1017964 26327262 PMC4536947

[B85] Mann C. A. Sterman M. Kaiser D. A. (1996). Suppression of EEG rhythmic frequencies during somato-motor and visuo-motor behavior. Int. J. Psychophysiol. 23, 1–7. doi: 10.1016/0167-8760(96)00036-0 8880361

[B86] Marin M.-F. Zsido R. Song H. Lasko N. Killgore W. D. S. Rauch S. . (2017). Skin conductance responses and neural activations during fear conditioning and extinction recall across anxiety disorders. JAMA Psychiatry 74, 622–631. doi: 10.1001/jamapsychiatry.2017.0329 28403387 PMC5539837

[B87] Menghini L. Gianfranchi E. Cellini N. Patron E. Tagliabue M. Sarlo M. (2019). Stressing the accuracy: wrist-worn wearable sensor validation over different conditions. Psychophysiology 56:e13441. doi: 10.1111/psyp.13441 31332802

[B88] Milad M. R. Quirk G. J. (2012). Fear extinction as a model for translational neuroscience: ten years of progress. Annu. Rev. Psychol. 63, 129–151. doi: 10.1146/annurev.psych.121208.131631 22129456 PMC4942586

[B89] Mishra A. Shukla A. Rana N. P. Dwivedi Y. K. (2020). From “touch” to a “multisensory” experience: the impact of technology interface and product type on consumer responses. Psychol. Mark. 38, 385–396. doi: 10.1002/mar.21436

[B90] Moldoveanu A. Mitru? O. Jinga N. Petrescu C. Moldoveanu F. Asavei V. . (2023). Immersive phobia therapy through adaptive virtual reality and biofeedback. Appl. Sci. 13:10365. doi: 10.3390/app131810365

[B91] Morey R. A. Haswell C. C. Stjepanović D. Dunsmoor J. E. LaBar K. S. (2020). Neural correlates of conceptual-level fear generalization in posttraumatic stress disorder. Neuropsychopharmacology 45, 1380–1389. doi: 10.1038/s41386-020-0661-8 32222725 PMC7297719

[B92] Mullen T. Kothe C. Chi Y. M. Ojeda A. Kerth T. Makeig S. . (2013). “Real-time modeling and 3D visualization of source dynamics and connectivity using wearable EEG,” in 2013 35th annual international conference of the IEEE engineering in medicine and biology society (EMBC) (Osaka: IEEE), 2184–2187. doi: 10.1109/EMBC.2013.6609968 24110155 PMC4119601

[B93] Munoz M. L. van Roon A. Riese H. Thio C. Oostenbroek E. Westrik I. . (2015). Validity of (ultra-)short recordings for heart rate variability measurements. PLoS ONE 10:e0138921. doi: 10.1371/journal.pone.0138921 26414314 PMC4586373

[B94] Nath R. K. Thapliyal H. (2021). Machine learning-based anxiety detection in older adults using wristband sensors and context feature. SN Comput. Sci. 2:359. doi: 10.1007/s42979-021-00744-z

[B95] Nazarova A. Teselkin D. Zajceva D. Obukhov A. (2023). Identification of patterns of anxiety-phobic disorders based on the analysis of encephalograms using virtual reality. AIP Conf. Proc. 2948:020008. doi: 10.1063/5.0165758

[B96] Nelson B. W. Low C. A. Jacobson N. Areán P. Torous J. Allen N. B. (2020). Guidelines for wrist-worn consumer wearable assessment of heart rate in biobehavioral research. NPJ Digit. Med. 3:90. doi: 10.1038/s41746-020-0297-4 32613085 PMC7320189

[B97] Obukhov A. Krasnyanskiy M. Volkov A. Nazarova A. Teselkin D. Patutin K. . (2023). Method for assessing the influence of phobic stimuli in virtual simulators. J. Imaging 9:195. doi: 10.3390/jimaging9100195 37888302 PMC10607658

[B98] Okazaki M. Kaneko Y. Yumoto M. Arima K. (2008). Perceptual change in response to a bistable picture increases neuromagnetic beta-band activities. Neurosci. Res. 61, 319–328. doi: 10.1016/j.neures.2008.03.010 18468708

[B99] Olivas G. Candela M. Ocampo J. Acosta L. Aguilar A. Delgado E. . (2021). “Detecting change in engineering interest in children through machine learning using biometric signals,” in 2021 machine learning-driven digital technologies for educational innovation workshop (Monterrey: IEEE), 1–7. doi: 10.1109/IEEECONF53024.2021.9733772

[B100] Ortiz-Espinoza A. Cebral-Loureda M. Hernandez-Morales J. Torres-Huitzil C. Ramírez-Moreno M. A. (2025). Real-time generative ai experience of the vanitas painting. Cogent Arts Humanit. 12:2591387. doi: 10.1080/23311983.2025.2591387

[B101] Pfurtscheller G. Lopes da Silva F. (1999). Event-related EEG/MEG synchronization and desynchronization: basic principles. Clin. Neurophysiol. 110, 1842–1857. doi: 10.1016/S1388-2457(99)00141-8 10576479

[B102] Picó-Pérez M. Alemany-Navarro M. Dunsmoor J. Radua J. Albajes-Eizagirre A. Vervliet B. . (2019). Common and distinct neural correlates of fear extinction and cognitive reappraisal: a meta-analysis of FMRI studies. Neurosci. Biobehav. Rev. 104, 102–115. doi: 10.1016/j.neubiorev.2019.06.029 31278951

[B103] Pineda-Hernández S. (2022). How to play under pressure: EEG monitoring of mental activation training in a professional tennis player. Physiol. Behav. 250:113784. doi: 10.1016/j.physbeh.2022.113784 35339575

[B104] Pirazzini G. Starita F. Ricci G. Garofalo S. di Pellegrino G. Magosso E. . (2023). Changes in brain rhythms and connectivity tracking fear acquisition and reversal. Brain Struct. Funct. 228, 1259–1281. doi: 10.1007/s00429-023-02646-7 37129622 PMC10250514

[B105] Piskorski J. Guzik P. (2007). Filtering poincaré plots. Comput. Methods Sci. Technol. 11, 39–48. doi: 10.12921/cmst.2005.11.01.39-48

[B106] Pope A. T. Bogart E. H. Bartolome D. S. (1995). Biocybernetic system evaluates indices of operator engagement in automated task. Biol. Psychol. 40, 187–195. doi: 10.1016/0301-0511(95)05116-3 7647180

[B107] Posada-Quintero H. F. Bolkhovsky J. B. (2019). Machine learning models for the identification of cognitive tasks using autonomic reactions from heart rate variability and electrodermal activity. Behav. Sci. 9:45. doi: 10.3390/bs9040045 31027251 PMC6523197

[B108] Posada-Quintero H. F. Chon K. (2020). Innovations in electrodermal activity data collection and signal processing: a systematic review. Sensors 20:479. doi: 10.3390/s20020479 31952141 PMC7014446

[B109] Prete G. Laeng B. Fabri M. Foschi N. Tommasi L. (2015). Right hemisphere or valence hypothesis, or both? The processing of hybrid faces in the intact and callosotomized brain. Neuropsychologia 68, 94–106. doi: 10.1016/j.neuropsychologia.2015.01.002 25575451

[B110] Ramírez-Moreno M. A. Carrillo-Tijerina P. Candela-Leal M. O. Alanis-Espinosa M. Tudón-Martínez J. C. Roman-Flores A. . (2021). Evaluation of a fast test based on biometric signals to assess mental fatigue at the workplace–a pilot study. Int. J. Environ. Res. Public Health Monterrey. 18:11891. doi: 10.3390/ijerph182211891 34831645 PMC8621458

[B111] Ramírez-Moreno M. A. Hernández-Mustieles M. A. Candela-Leal M. O. Tudón-Martinez J. C. Lozoya-Santos J. d. J. (2026). Workplace Measures of Mental Fatigue. London: Elsevier, 209–218. doi: 10.1016/B978-0-443-24080-5.00005-9

[B112] Rantanen A. Laukka S. Siipo A. Tiinanen S. Tarvainen M. P. Kortelainen J. . (2021). Verbalization has regulatory influences on autonomic activity during recall of unpleasant experience. Speech Commun. 126, 1–8. doi: 10.1016/j.specom.2020.11.002

[B113] Ray W. J. Cole H. W. (1985). EEG alpha activity reflects attentional demands, and beta activity reflects emotional and cognitive processes. Science 228, 750–752. doi: 10.1126/science.3992243 3992243

[B114] Reeves R. Elliott A. Curran D. Dyer K. Hanna D. (2021). 360° video virtual reality exposure therapy for public speaking anxiety: a randomized controlled trial. J. Anxiety Disord. 83:102451. doi: 10.1016/j.janxdis.2021.102451 34304028

[B115] Rezaei S. Parmeh N. Rajabpour V. Rahimi F. (2025). A machine learning approach for detection of claustrophobic brain activity in electroencephalography. Sci. Rep. 15:35185. doi: 10.1038/s41598-025-19167-4 41062700 PMC12508191

[B116] Ribas V. R. Ribas R. G. Nóbrega J. d. A. Nóbrega M. V. d. Espécie J. A. d. A. Calafange M. T. . (2018). Pattern of anxiety, insecurity, fear, panic and/or phobia observed by quantitative electroencephalography (QEEG). Dement. Neuropsychol. 12, 264–271. doi: 10.1590/1980-57642018dn12-030007 30425790 PMC6200158

[B117] Rihs T. A. Michel C. M. Thut G. (2007). Mechanisms of selective inhibition in visual spatial attention are indexed by alpha-band EEG synchronization. Eur. J. Neurosci. 25, 603–610. doi: 10.1111/j.1460-9568.2007.05278.x 17284203

[B118] Riva G. Botella C. Baños R. Mantovani F. García-Palacios A. Quero S. . (2015). Presence-Inducing Media for Mental Health Applications. Cham: Springer International Publishing. 283–332. doi: 10.1007/978-3-319-10190-3_12

[B119] Riva G. Mantovani F. Capideville C. S. Preziosa A. Morganti F. Villani D. . (2007). Affective interactions using virtual reality: the link between presence and emotions. Cyberpsychol. Behav. 10, 45–56. doi: 10.1089/cpb.2006.9993 17305448

[B120] Romano J. Kromrey J. D. Coraggio J. Skowronek J. (2006). “Appropriate statistics for ordinal level data: should we really be using *t*-test and cohen's d for evaluating group differences on the NSSE and other surveys?” in Annual meeting of the Florida association of institutional research (FAIR) (Naples, FL: Florida Association of Institutional Research).

[B121] Romo-De León R. Cham-Pérez M. L. L. Elizondo-Villegas V. A. Villarreal-Villarreal A. Ortiz-Espinoza A. A. Vélez-Saboyá C. S. . (2024). EEG and physiological signals dataset from participants during traditional and partially immersive learning experiences in humanities. Data 9:68. doi: 10.3390/data9050068

[B122] Romo-De León R. Cham-Pérez M. L. L. Elizondo-Villegas V. A. Villarreal-Villarreal A. Ortiz-Espinoza A. A. Vélez-Saboyá C. S. . (2026). Neurophysiological assessment of biometric patterns during semi-immersive and traditional learning experiences in the humanities. Front. Hum. Neurosci. 20:1692599. doi: 10.3389/fnhum.2026.1692599 41710215 PMC12909478

[B123] Rousselet G. A. Pernet C. R. Wilcox R. R. (2017). Beyond differences in means: robust graphical methods to compare two groups in neuroscience. Eur. J. Neurosci. 46, 1738–1748. doi: 10.1111/ejn.13610 28544058

[B124] Russo S. Tibermacine I. E. Tibermacine A. Chebana D. Nahili A. Starczewscki J. . (2024). Analyzing EEG patterns in young adults exposed to different acrophobia levels: a VR study. Front. Hum. Neurosci. 18:1348154. doi: 10.3389/fnhum.2024.1348154 38770396 PMC11102978

[B125] Sadhukhan D. Pal S. Mitra M. (2018). “PPG noise reduction based on adaptive frequency suppression using discrete fourier transform for portable home monitoring applications,” in 2018 15th IEEE India council international conference (INDICON) (Coimbatore), 1–6. doi: 10.1109/INDICON45594.2018.8987004

[B126] Sauseng P. Klimesch W. Stadler W. Schabus M. Doppelmayr M. Hanslmayr S. . (2005). A shift of visual spatial attention is selectively associated with human EEG alpha activity. Eur. J. Neurosci. 22, 2917–2926. doi: 10.1111/j.1460-9568.2005.04482.x 16324126

[B127] Schipke J. Pelzer M. Arnold G. (1999). Effect of respiration rate on short-term heart rate variability. J. Clin. Basic Cardiol. 2, 92–95.

[B128] Schmidt P. Reiss A. Duerichen R. Marberger C. Van Laerhoven K. (2018). “Introducing WESAD, a multimodal dataset for wearable stress and affect detection,” in Proceedings of the 20th ACM international conference on multimodal interaction, ICMI '18 (New York, NY: Association for Computing Machinery), 400–408. doi: 10.1145/3242969.3242985

[B129] Schubert T. W. (2003). The sense of presence in virtual environments: a three-component scale measuring spatial presence, involvement, and realness. Zeitschrift für Medienpsychologie 15, 69–71. doi: 10.1026//1617-6383.15.2.69

[B130] Seuling P. D. Czernin N. S. Schiele M. A. (2024). Virtual reality exposure therapy in the treatment of public speaking anxiety and social anxiety disorder. Neurosci. Appl. 3:104074. doi: 10.1016/j.nsa.2024.104074 40656084 PMC12244110

[B131] Shaffer F. Ginsberg J. P. (2017). An overview of heart rate variability metrics and norms. Front. Public Health 5:258. doi: 10.3389/fpubh.2017.00258 29034226 PMC5624990

[B132] Shaffer F. McCraty R. Zerr C. L. (2014). A healthy heart is not a metronome: an integrative review of the heart's anatomy and heart rate variability. Front. Psychol. 5:1040. doi: 10.3389/fpsyg.2014.01040 25324790 PMC4179748

[B133] Shaffer F. Meehan Z. M. Zerr C. L. (2020). A critical review of ultra-short-term heart rate variability norms research. Front. Neurosci. 14:594880. doi: 10.3389/fnins.2020.594880 33328866 PMC7710683

[B134] Slater M. (1999). Measuring presence: a response to the witmer and singer presence questionnaire. Presence Teleoperators Virtual Environ. 8, 560–565. doi: 10.1162/105474699566477

[B135] Sperl M. F. Wroblewski A. Mueller M. Straube B. Mueller E. M. (2021). Learning dynamics of electrophysiological brain signals during human fear conditioning. NeuroImage 226:117569. doi: 10.1016/j.neuroimage.2020.117569 33221446

[B136] Staib M. Castegnetti G. Bach D. R. (2015). Optimising a model-based approach to inferring fear learning from skin conductance responses. J. Neurosci. Methods 255, 131–138. doi: 10.1016/j.jneumeth.2015.08.009 26291885 PMC4612446

[B137] Stiedl O. Hager T. (2017). “Cardiovascular conditioning: neural substrates,” in Reference Module in Neuroscience and Biobehavioral Psychology, (Amsterdam: Elsevier). doi: 10.1016/B978-0-12-809324-5.00279-0

[B138] Thayer J. F. Åhs F. Fredrikson M. Sollers J. J. Wager T. D. (2012). A meta-analysis of heart rate variability and neuroimaging studies: implications for heart rate variability as a marker of stress and health. Neurosci. Biobehav. Rev. 36, 747–756. doi: 10.1016/j.neubiorev.2011.11.009 22178086

[B139] Thomas B. Claassen N. Becker P. Viljoen M. (2019). Validity of commonly used heart rate variability markers of autonomic nervous system function. Neuropsychobiology 78, 14–26. doi: 10.1159/000495519 30721903

[B140] Toma E. Balan O. Lambru C. Moldoveanu A. Moldoveanu F. (2020). “Ophiophobia 3d-a game for treating fear of snakes,” in Proceedings of the 2020 IEEE 10th international conference on intelligent systems, IS 2020 (Varna: IEEE). doi: 10.1109/IS48319.2020.9200189

[B141] Tomarken A. J. Davidson R. J. Henriques J. B. (1990). Resting frontal brain asymmetry predicts affective responses to films. J. Pers. Soc. Psychol. 59, 791–801. doi: 10.1037/0022-3514.59.4.791 2254854

[B142] Tychkov A. Y. Chernyshov D. S. Bofonova N. S. Churakov P. P. Yuldashev Z. M. Gorbunov V. N. . (2023). Spectral analysis of the EEG of subjects with anxious-phobic disorders in a virtual reality environment. Biomed. Eng. 56, 429–434. doi: 10.1007/s10527-023-10250-6

[B143] Ugalde-Canitrot A. García-Fernández M. Gómez-Moroney A. Saceda J. García de Leonardo Mena C. Martín D. M. (2025). Ictal fear in children: localizing value and insights into the mechanisms of fear in the developing brain. Pediatr. Neurol. 169, 82–90. doi: 10.1016/j.pediatrneurol.2025.05.010 40472589

[B144] van Gent P. Farah H. Nes N. Arem B. (2018). “Heart rate analysis for human factors: development and validation of an open source toolkit for noisy naturalistic heart rate data,” in 2018 6th humanist conference, (The Hague).

[B145] Veeranki Y. R. Ganapathy N. Swaminathan R. Posada-Quintero H. F. (2024). Comparison of electrodermal activity signal decomposition techniques for emotion recognition. IEEE Access 12, 19952–19966. doi: 10.1109/ACCESS.2024.3361832

[B146] Villafaina S. Castro M. A. Pereira T. Carvalho Santos A. Fuentes-García J. P. (2021a). Neurophysiological and autonomic responses of high and low level chess players during difficult and easy chess endgames - a quantitative EEG and HRV study. Physiol. Behav. 237:113454. doi: 10.1016/j.physbeh.2021.113454 33971186

[B147] Villafaina S. Collado-Mateo D. Cano-Plasencia R. Gusi N. Fuentes J. P. (2019). Electroencephalographic response of chess players in decision-making processes under time pressure. Physiol. Behav. 198, 140–143. doi: 10.1016/j.physbeh.2018.10.017 30389477

[B148] Villafaina S. Fuentes-García D. J. P. Gusi N. Tornero-Aguilera J. F. Clemente-Suárez V. J. (2021b). Psychophysiological response of military pilots in different combat flight maneuvers in a flight simulator. Physiol. Behav. 238:113483. doi: 10.1016/j.physbeh.2021.113483 34097973

[B149] Wannemueller A. Margraf J. Busch M. Jöhren H.-P. Suchan B. (2024). More than fear? brain activation patterns of dental phobic patients before and after an exposure-based treatment. J. Neural Transm. 131, 393–404. doi: 10.1007/s00702-024-02754-6 38424282 PMC11016127

[B150] Webb S. (2020). Interpreting kant in education: dissolving dualisms and embodying mind - introduction. J. Philos. Educ. 54, 1494–1509. doi: 10.1111/1467-9752.12529

[B151] Wehrwein E. A. Orer H. S. Barman S. M. (2016). Overview of the anatomy, physiology, and pharmacology of the autonomic nervous system. Compr. Physiol. 6, 1239–1278. doi: 10.1002/j.2040-4603.2016.tb00714.x 27347892

[B152] Witmer B. G. Singer M. J. (1998). Measuring presence in virtual environments: a presence questionnaire. Presence: Teleop. Virt. Environ. 7, 225–240. doi: 10.1162/105474698565686

[B153] Yaşgüçlükal M. A. Makhalova J. Carron R. Bartolomei F. (2021). Ictal fear during parietal seizures. Epileptic Disord. 23, 793–798. doi: 10.1684/epd.2021.1321 34588160

[B154] Yu X. Lu J. Liu W. Cheng Z. Xiao G. (2024). Exploring physiological stress response evoked by passive translational acceleration in healthy adults: a pilot study utilizing electrodermal activity and heart rate variability measurements. Sci. Rep. 14:11349. doi: 10.1038/s41598-024-61656-5 38762532 PMC11102551

